# Cathepsin K regulates the tumor growth and metastasis by IL-17/CTSK/EMT axis and mediates M2 macrophage polarization in castration-resistant prostate cancer

**DOI:** 10.1038/s41419-022-05215-8

**Published:** 2022-09-22

**Authors:** Ning Wu, YouZhi Wang, KeKe Wang, BoQiang Zhong, YiHao Liao, JiaMing Liang, Ning Jiang

**Affiliations:** 1 Department of Urology, Tianjin Institute of Urology, The Second Hospital of Tianjin Medical University, Tianjin, 300211 PR China; 2 Tianjin Medical University Cancer Institute and Hospital, Tianjin Medical University, Tianjin, 300060 China

**Keywords:** Tumour immunology, Prostate cancer, Prostate cancer

## Abstract

A common stage of advanced prostate cancer is castration-resistant prostate cancer (CRPC), greater understanding of which is required in order to address and solve the clinically difficult challenge. Cathepsin K (CTSK) is a cysteine protease that usually has a strong activity of degrading extracellular matrix and is related to osteoclast-mediated bone destruction. However, the mechanism of CTSK-regulation in CRPC is still unclear to us. The current study aimed to analyze the expression of differentially expressed genes (DEGs) in patient samples (from localized PC and CRPC). Interestingly, we found that CTSK to be significantly up-regulated in CRPC. Through further signal pathway enrichment analysis, we found that the IL-17 signaling pathway to be highly correlated with CTSK. The oncogenic functions of CTSK and IL-17 in CRPC were proven by a series of in vivo and in vitro experiments. Possible downstream molecules of CTSK were investigated, which could serve as control elements to regulate the expression of EMT, thereby facilitating the metastasis and excessive proliferation of PC cells. Expression of CTSK was related to high concentration of M2 tumor-associated macrophages (TAMs) M2 in CRPC. A CTSK-mediated feedback circuit between TAMs and CRPC tissues was indicated in the process of transfer, proving the possibility of CTSK could be use as an available therapeutic target for CRPC.

## Introduction

Prostate cancer (PC) is a primary cause of mortality in patients with cancer, accounting for ~1–2% of deaths in men worldwide [1]. Although the authoritative therapeutic method for metastatic PC (mPC) is androgen deprivation therapy (ADT), the disease progresses to CRPC in majority of patients within 2 years and is associated with a grim prognosis [2, 3]. Therefore, identification of targets and key pathways for the treatment of CRPC is urgently required.

CTSK belongs to the peptidase C1 family, mainly participating in bone resorption and remodeling [4, 5]. It is also involved in keratinocyte differentiation and prohormone activation [5]. CTSK has been found in different types of cancer, including PC, breast cancer, and colorectal cancer. Interestingly, variable expression of CTSK has been observed in PC samples and non-osseous metastases; while expression in bone metastases was significantly higher than in primary PC, that in normal prostate tissues was negative [6, 7]. Interleukin-17 (IL-17) is a critical pro-inflammatory cytokine that is mainly secreted by immune cells, including γδ T cells, NK cells, and TH17 cells [8]. In fact, IL-17 not only plays a vital role in autoimmune and inflammatory diseases, but also has been proven to accelerate the development of PC, skin cancer, colon cancer, breast cancer, and lung cancer [9–11]. The IL-17RA/IL-17RC receptor complex, as the main active place of IL-17, recruits NF-κB activator 1 (Act1) [12]. Tumor necrosis factor receptor-associated factor 6 is enabled by Act1, which then activates IkB kinase and transforming growth factor-β-activated kinase 1, leading to the activation of NF-κB that further triggers the transcription of all kinds of cytokines and chemokines, such as IL-6, C-X-C motif ligand 1, and IL-1β [13]. The IL-17-regulated factors promote the progression of cancer by increasing angiogenesis and reducing apoptosis, increasing cell proliferation, and improving the immunologic tolerance of microenvironment [14, 15].

In this study, we proved CTSK as a vital protein, whose level can increase due to the up-regulation of IL-17A expression and whose transfer capability can be further enhanced. The effects of CTSK and IL-17/CTSK/EMT axis on PC tissues and cells were further verified in this study. The above axis was found to affect both androgen-independent and androgen-dependent cells, and had more significant effects on DU145 cell lines. Finally, the influence of CTSK and IL-17A on tumor-associated macrophages M2 in PC tissues was verified by database analysis and appropriate experiments.

## Materials and methods

### Data collection

We screened datasets from Gene Expression Omnibus (GEO), and The Cancer Genome Atlas to explore [16]. In this article, we selected datasets of localized samples and CRPC samples from the GEO database. Finally, GSE32269, GSE32982, and GSE70770 met our inclusion criteria. Details of these three datasets are listed in the Supplementary Table. We used the SVA package in R language to combine the data into a database containing 276 samples, of which 45 were CRPC samples and 231 were localized samples.

### Screening and identification of the differentially expressed genes (DEGs)

We compared the protein expression data of the localized samples with those of metastatic samples using limma package of R (3.48.3) to identify the significant DEGs (|Log2FC | > 1.3, adjusted *p* value < 0.05) [17]. Volcano plots and heatmaps were used to characterize the DEGs, and the KEGG pathway database was used to choose the signal pathways in DEGs enrichment [18, 19].

### WGCNA

At first, we selected DEGs between localized and metastatic samples from the combined set of GSE32269, GSE32982, and GSE70770. Thereafter, we executed the weighted gene co-expression network analysis (WGCNA) (1.70-3) on the basis of DEGs in R. The DEGs were hierarchically clustered into eight gene modules when the *β* value was defined as 6.

### Survival analysis

We downloaded the data from GSE32269 dataset and analyzed the ROC curve in SPSS to identify the specificity and sensitivity. Survival analysis was performed on the GSE16560 dataset.

### Statistical analysis

Continuous data were described using mean ± standard deviation or median (interquartile range). The *t*-test was used to compare normal data while the Mann–Whitney *U* test was used to compare non-normal data. Spearman correlation analysis was used to analyze the correlations in the cross-sectional study. The receiver operating characteristic (ROC) curve and area under the curve were used to calculate the best predictive cut-off value; *p* values < 0.05 were considered statistically significant. Statistical analyses of the data were performed using GraphPad Prism version 8.0 and SPSS version 25.0.

### Serum sCD206 levels of patients with PC

All serum samples were routinely collected from patients before administering treatments, during hospitalization, and stored at −80 °C. The concentrations of sCD206 were measured using commercial enzyme-linked immunosorbent assay kits (Human MMR ELISA Kits, RayBiotech, Norcross, GA). First, 100 μl of standard solutions or samples was added to each and incubated for 2.5 h. After four washes, 100 μl of prepared biotin antibodies was added to each well. After 1-h incubation, 100 μl of prepared streptavidin solution was added and incubated for 45 min. The mixture was washed four times, and 100 μl of TMB one-step substrate reagent was added to each well and incubated for 30 min, followed by another four rounds of washing. Finally, 50 μl of stop was added to each well.

### Patients

Prostate tissue specimens, used in this study, were surgical specimens from patients with PC haying complete clinicopathological data. ADPC specimens were acquired by radical prostatectomy, and BPH/CRPC specimens were acquired by transurethral resection of the prostate. These samples were paraffin-embedded and subjected to IHC with standard DAB staining protocols. All tissue samples were obtained from patients with PC, who had undergone surgical operation in the Second Affiliated Hospital of Tianjin Medical University (Tianjin, China), and were inspected by three qualified pathologists to acquire accurate grades. The main site of metastasis was bone tissues in patients with mPC.

### Cell lines

Benign prostatic hyperplasia cells (BPH) and various PC cell lines (22Rv1, C4-2, PC3, LNCaP, and DU145) were acquired from ATCC. The cells were cultured in RPMI 1640 medium supplemented with 10% fetal bovine serum and 1% penicillin/streptomycin in a humidified environment containing 5% CO_2_ at 37 °C.

### Western blot (WB)

Proteins from BPH, 22Rv1, C4-2, PC3, LNCaP, DU145, and different carcinomas were extracted using PMSF and RIPA. BCA kit was used to detect the concentration of different proteins. Protein samples were separated by SDS-polyacrylamide gel electrophoresis in 10% acrylamide gel and then transferred to polyvinylidene fluoride membrane. Next, the was blocked with skimmed milk powder (5%) and incubated with primary antibodies [GAPDH (1:1000) (ab9485), AR-V7 (1:1000) (ab198394), vimentin (1:1000) (ab92547), β-catenin (1:1000) (ab32572), β-tubulin (1:1000) (ab78078), E-cadherin (1:1000) (ab40772), claudin-1 (1:1000) (ab211737), slug (1:1000) (ab180714), twist (1:1000) (ab175430), snail (1:1000) (ab180714)] at 4 °C overnight. The membrane was washed twice with PBS, and incubated with anti-mouse IgG/anti-rabbit IgG at normal temperature for 1 h. The membrane was washed twice with PBS once again. Finally, wb automatic chemiluminescence imaging system was used to detect the bands.

### Flow cytometry

For flow cytometry analysis, the tumor mass was dissociated into single cells. Prior to antibody staining, red blood cells were removed with ammonium chloride-potassium lysis buffer for 3 min at room temperature, followed by incubation or staining with cell surface antibody [APC anti-mouse CD206 (BioLegend)] for 30 min on ice. The cells were then washed twice and re-suspended in FACS buffer. Flow cytometry was performed using a CytoFLEX flow cytometer (Beckman Coulter), and the resulting data were analyzed using CytExpert software.

### IHC

After clinical and animal laboratory surgery, we obtained human tissue specimens and mouse tumor specimens, which were then fixed with formalin. We prepared pathological sections of the specimens by freezing, paraffin fixing, and sectioning. Next, we put the pathological sections into the oven at 60 °C for 60 min, dewaxed the slices in xylene, and used graded alcohol for rehydration. Thereafter, we used PBS to wash the pathological sections twice, and used citric acid buffer to recover the pathological sections (7 min on high fire and 10 min on medium fire). The pathological sections were washed twice with PBS, and endogenous peroxidase was added to sections for 20 min. Finally, primary antibody was added to the pathological section and left in the refrigerator at 4 °C overnight. The next day, we uesd secondary antibody to detect after washing the pathological sections with PBS twice. DAB chromogen was used for detection, and hematoxylin was used for redyeing it after washing with tap water. After dehydration, the plates were sealed with neutral gum and photographed under a microscope.

### Wound healing

We seeded PC cells into 6-well plates. Next, we drew a straight line in the middle of the plates with the tip of the 10-μl microsphere after transfecting negative control siRNA, negative control siRNA + IL-17A, CTSK siRNA, and CTSK siRNA + IL-17A into PC cells (after 24 h). Thereafter, we washed the plates twice with PBS and cultured the cells in a cell incubator for 24–72 h. Photographs were taken under a microscope at 0, 24, and 72 h, respectively.

### Clone formation assay

We seeded digestive cells (2.0 × 10^3^ DU145 cells, 2.0 × 10^3^ LNCaP cells) into a 6-well plate. Then, we transfected negative control siRNA, negative control siRNA + IL-17A, CTSK siRNA, or CTSK siRNA + IL-17A into the cells after 24 h. After 1–2 weeks of culturing the PC cells, the plates were washed twice with PBS. Next, we fixed the cells with paraformaldehyde and and used PBS to wash the plates again twice. Finally, the cells were stained with crystal violet for 0.5 h, and the plates were washed twice with PBS and dried thereafter.

### Transwell migration

We transfected CTSK-siRNA or negative control siRNA into DU145 or LNCaP cells, respectively. We added 2 × 10^4^ cells and 1640 (10% FBS) to the top of transwell insert, and 1640 (10% FBS) to the bottom chamber. Then, we cultivated the cells for 48 h at 37 °C, and used PBS to wash the chambers twice. Next, we fixed the cells with paraformaldehyde and used PBS to wash the chambers twice. Finally, the cells were stained with crystal violet for 1 h.

### Tumor xenografts mouse model

Male mice were injected with 2 × 10^6^ PC3 cells, suspended in 150 μl of Matrigel and 1640 medium, under the skin of the abdomen in control, control + IL-17A, shCTSK, and shCTSK + IL-17A groups. Tumor volume data were collected for at least 2 weeks, being measured at the same time every day. Finally, the mice were sacrificed and weight of the tumor were measured with precision. Parts of the fresh specimens were examined by flow cytometry to verify the immune-related indicators. Rest of the mouse tumors were fixated with paraformaldehyde, and immunohistochemical staining was performed for markers of CTSK, β-catenin, vimentin and E-cadherin. All procedures involving mice were approved by the University Committee on Use and Care of Animals at the Tianjin Medical University and met all regulatory standards.

## Results

### Over-expression of CTSK-associated IL-17A in castration-resistant prostate cancer

**Fig. 1:**
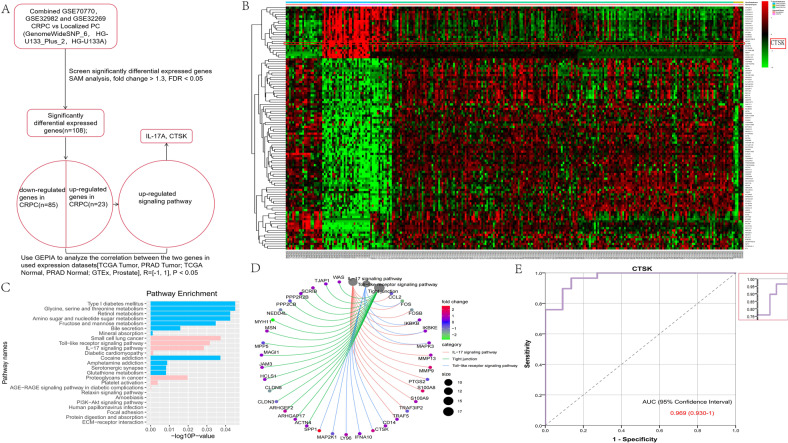
Over-expression of CTSK-associated IL-17A in castration-resistant prostate cancer. **A** Principle description of the work procedure used to study the “IL-17A/CTSK” axis in CRPC. The expression profiles data of various types of prostate cancer (#GSE70770, GSE32982 and GSE32269). **B** Heatmap of 23 up-regulated genes and 85 down-regulated genes mined from GSE70770, GSE32982 and GSE32269. The lower bar shows different groups, the pink one represents the group of CRPC and the orange red one represents the group of localized PC. **C** The bar chart shows KEGG pathway enrichment data for DEGs. Pink represents up-regulated pathways and blue represents down-regulated pathways. **D** Circle diagram was adopted for further exploration to clarify the expression genes involved in the three significantly up-regulated pathways. **E** Estimate the therapeutic values of CTSK by analyzing the ROC curve (*p* < 0.001).

Our screening method was designed by combining the a set of databases of gene expression (Fig. 1A) to investigate the “IL-17A-CTSK” regulatory axis in CRPC. It aimed to analyze the DEGs between localized PC and CRPC tissues using an incorporative dataset having three microarray datasets (GSE70770, GSE32982 and GSE32269) (Figs. 1B and S1A, B). A total of 108 DEGs (85 down-regulated genes and 23 up-regulated genes) were identified in the incorporative cohort. CTSK was found to be up-regulated in the CRPC tissues (Fig. 1B). KEGG pathway enrichment analyses were used to find and characterize the possible functions of the selected DEGs from the integrated dataset. According to KEGG analysis, pathways associated with CRPC specific up-regulated expression were enriched, including the interleukin (IL)-17 signaling pathway, toll-like receptor signaling pathway, small cell lung cancer, and proteoglycans in cancer (Fig. 1C). Aimed at clarifying the expression of genes involved in the three significantly up-regulated pathways, the circle diagram and Gene Expression Profiling Interactive Analysis were adopted for further exploration. As a result, IL-17A was found to be correlated with CTSK, RFTN1, ARHGEF2 and MMP9 (Figs. 1D and S1C–F). Moreover, we utilized ROC curve to assess the potential values of CTSK in the GSE32269 datasets; as depicted in Fig. 1E, it was 0.969 (with a 95% fiducial interval of 0.930–1). Collectively, CTSK was shown to be linked to IL-17A, and may act as its downstream molecule. Over-expression of CTSK-associated IL-17A in castration-resistant PC predicted poor prognosis.

### Higher CTSK expression in metastatic prostate cancer/castration-resistant prostate cancer and the associated poor prognosis

**Fig. 2:**
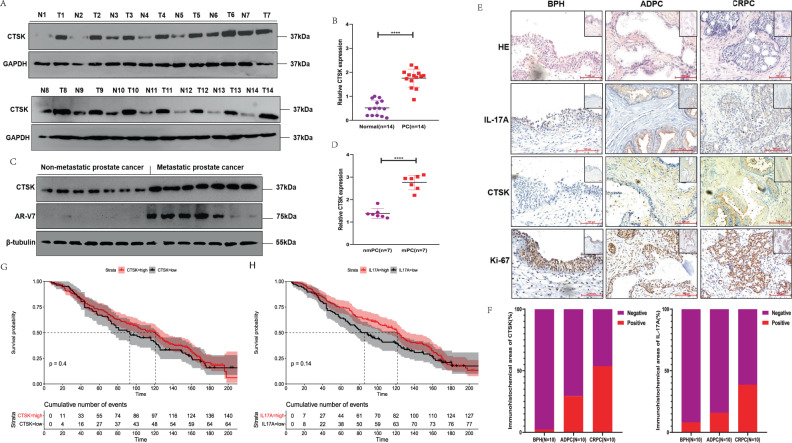
Higher CTSK expression in metastatic prostate cancer/castration-resistant prostate cancer and the associated poor prognosis. **A** WB assay to examine the CTSK protein expression in CRPC tissues (T) and normal prostate tissues (N), GAPDH protein served as control. **B** The scatter diagram represents the relative CTSK expression in CRPC and normal tissues. **C** WB assay to examine the CTSK and AR-V7 protein expression in non-metastatic (nmPC) and metastatic (mPC) cancer tissues, β-tubulin protein served as control. **D** The scatter diagram represents the relative CTSK expression in nmPC and mPC tissues. **E** Comparing CTSK and IL-17A expression in BPH, ADPC, and CRPC groups by IHC staining. **F** The histogram represents comparison of the expression of CTSK and IL-17A in three kinds of groups. **G** Univariate analyses (log-rank) and Kaplan–Meier survival curves for PC patients with distinct CTSK expression levels. **H** Univariate analyses (log-rank) and Kaplan–Meier survival curves for PC patients with distinct IL-17A expression levels. Data are shown as mean ± s.e.m. *p < 0.05.

In Fig. 2A, B, over-expression of CTSK in 14 PC tissues, compared to that in matched non-tumor tissues, has been shown, based on CTSK measurement in 40 patients with PC. According to the analyzed data, CTSK and AR-V7 tended to show higher expression in mPC (*n* = 7) than in non-mPC tissues (*n* = 7) (Fig. 2C, D). Moreover, to assess the clinical implication of CTSK and IL-17A, we conducted IHC experiments in ten BPH tissues, ten androgen-dependent PC (ADPC), and ten CRPC tissues. CTSK was overexpressed in 53% (8/10) of the CRPC tissues, although the expression of CTSK was only observed in 29% (5/10) of ADPC samples and in 2% (10/10) of BPH samples. Similarly, IL-17A was overexpressed in 39% (8/10) of CRPC tissues, while CTSK was expressed only in 16% (5/10) of ADPC samples and in 8% (10/10) of BPH samples (Fig. 2E, F). Finally, based on of a published PC dataset (GSE16560, *n* = 281), we performed a Kaplan–Meier survival analysis; results showed that patients have a worse prognosis when they have high expression of CTSK and IL-17A (Fig. 2G, H).

### Effect of IL-17A on the growth of prostate cancer cells via its influence on the expression of CTSK both in vivo and in vitro

**Fig. 3:**
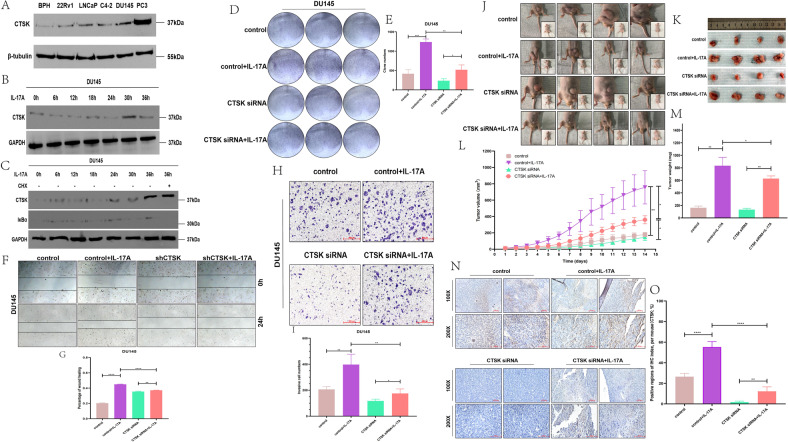
Effect of IL-17A on the growth of prostate cancer cells via its influence on the expression of CTSK both in vivo and in vitro. **A** WB assay to examine the CTSK protein expression in PC cells, β-tubulin protein served as control. **B** Treated with 15 ng/ml restructuring human IL-17A in DU145 cells for 0, 6, 12, 18, 24, 30, and 36 h. WB assay to examine the CTSK protein expression, GAPDH protein served as control. **C** WB assay to examine the CTSK/IκBα protein expression, GAPDH protein served as control. Treated with 15 ng/ml restructuring human IL-17A in DU145 cells for 0–36 h and one group is added 10 µg/ml CHX at 24 h and collected at 36 h. **D** DU145 cells are treated with control siRNA, IL-17A, CTSK siRNA, CTSK siRNA + IL-17A, and conducted colony formation assay. **E** Quantization of **D**. **F** Wound healing assay in DU145 cells transfected with negative control siRNA, IL-17A, CTSK siRNA, CTSK siRNA + IL-17A. **G** Quantization of **F**. **H** Transwell assay in DU145 cells transfected with negative control siRNA, IL-17A, CTSK siRNA, CTSK siRNA + IL-17A. **I** Quantization of **H**. **J**, **K** DU145 cells treated with negative control siRNA, IL-17A, CTSK siRNA, CTSK siRNA + IL-17A are injected subcutaneously into the abdomen of mice. The effect of negative control siRNA, IL-17A, CTSK siRNA, CTSK siRNA + IL-17A on the growth of PCa. **L** Measuring tumor sizes of mice daily for 2 weeks when the tumor is 1.5–2 mm in diameter. **M** Quantification of tumors in different groups of mice in **L**. **N**, **O** Comparing CTSK expression in negative control siRNA, IL-17A, CTSK siRNA, CTSK siRNA + IL-17A groups by IHC staining.

When CTSK expression levels of different PC cell lines were compared by western blot assays, CTSK in PC cell lines was significantly higher than that in BPH (Figs. 3A and S2A). CTSK expression is known to be to IL-17A, based on previous studies. Therefore, we hypothesized that CTSK could be a key gene downstream of IL-17A. During the experimental and investigation period, DU145 and PC3 cell lines were androgen-independent cell lines, and had been considered as CRPC cell lines by default in most literatures and experiments [20–22]. We treated DU145 cells with 15 ng/ml restructuring human IL-17A in for 6, 12, 18, 24, 30, and 36 h. CTSK protein was found to be enhanced by inducing IL-17A, and the protein levels started to change around 6–36 h in the DU145 cells (Figs. 3B and S2B). Additionally, we speculated CTSK protein to be relatively stable. Therefore, we added 10 µg/ml cycloheximide to one group of cells at 24 h of treatment with 15 ng/ml restructuring human IL-17A. Results showed that the levels of CTSK protein in CHX-untreated group and CHX-treated group to be similar at 36 h in DU145 cells (Figs. 3C and S2C, D). Similar findings were established with a well-known stable protein GAPDH. Hence, the concentrations of CHX was considered effective, since the levels of IκBα protein were reduced with 10 µg/ml CHX. We inoculated PC cells at a low density (2 × 10^3^ cells/chamber). After 7–14 days, the PC cells formed obvious colonies. The colony forming capacity of DU145 cells was prominently promoted in the increased-IL-17A group. Similarly, the ability of DU145 cells to colonize was reduced dramatically when they were treated with CTSK siRNA. However, despite the treatment of DU145 cells with CTSK siRNA, we found the colonizing ability of DU145 cell to be promoted upon increasing IL-17A (Fig. 3D, E). The effect of different levels of CTSK on the migration ability of DU145 cells were observed next, and we found that the IL-17A group promoted the migration ability remarkably compared to the remaining three groups in DU145 cell lines (Fig. 3F, G) and LNCaP cell lines (Fig. S3A, B). However, the differences were not obvious between the CTSK siRNA group and the CTSK siRNA + IL-17A group in LNCaP cell lines (Fig. S3A, B). Previous studies had reported that CTSK shows high expression in tumor cells that are highly invasive. Compared to the control group (Fig. 3H, I), the ability of invasion and migration in DU145 cell lines was found to be increased drastically in the IL-17A group, decreased drastically in the CTSK siRNA group, and promoted back when IL-17A was increased again. However, the invasion and migration ability of LNCaP cells did not change when IL-17A was increased again (Fig. S3C, D).

Furthermore, PC3 cells (2 × 10^6^) were transfected with either control siRNA, restructuring human IL-17A, CTSK siRNA, or CTSK siRNA + IL-17A, and inoculated under the abdomen of nude mice that were ~5 weeks old. When the tumor was 1.5–2 mm in diameter, we began to measure its sizes daily for 14 days (Fig. 3L). At 2 weeks, the nude mice were sacrificed under sterile conditions on an animal laboratory workbench. The weight/volume of IL-17A-over-expression group was distinctly different from that of the control groups (Fig. 3J–M). And the tumor weight/volume of CTSK siRNA group of mice were the smallest when compared with the rest of three groups (Fig. 3J–M). Next, the immunohistochemical test was adopted to verify the tumor in mice, and the results were same as above. IL-17A positive regions of the control + IL-17A group and CTSK siRNA + IL-17A group were significantly higher than the control group and CTSK siRNA group (Fig. S4).

The CTSK-positive regions in the IL-17A group were remarkably increased and those in the CTSK siRNA group were significantly decreased compared to that in the control groups; however, re-addition of IL-17A promoted the increased expression of CTSK again (Fig. 3N, O).

### Enhancement of EMT by IL-17A via CTSK induction

**Fig. 4:**
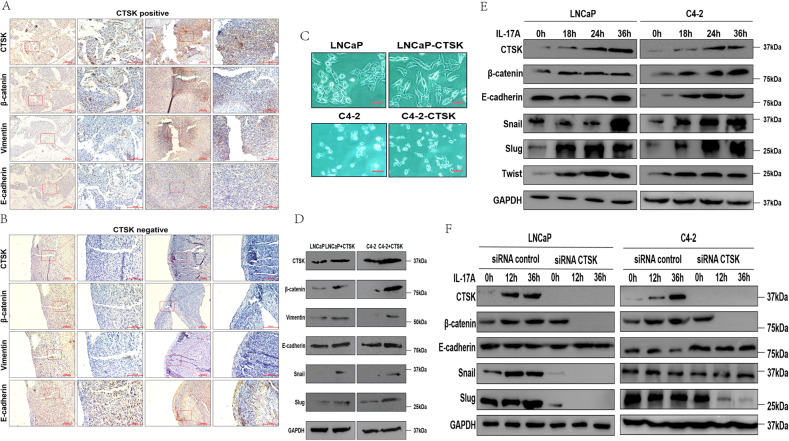
Enhancement of EMT by IL-17A via CTSK induction. **A** Subcutaneous tumor tissue of mice is stained for EMT markers (β-catenin, vimentin, E-cadherin). The mice tumor tissues express excessive CTSK. **B** Subcutaneous tumor tissue of mice is stained for EMT markers (β-catenin, vimentin, E-cadherin). The mice tumor tissues express a small amount of CTSK. **C** Phase-contrast photomicrographs of human prostate cancer cells in monolayer culture. **D**–**F** Western blot analysis of EMT markers in human prostate cancer cells.

Based on previous studies, CTSK, ZEB1, STC1, SPARC, and S100A6 proteins are known to be expressed in various and specific patterns in human PC [23]. Some studies have shown that in subsets of tumor epithelia, stromally expressed molecules exist, indicating that they are potential markers of EMT [24, 25]. First, we verified the different EMT indicators in the tissues of patients with PC and tumor tissues of animal models by IHC assay; results of IHC indicators in animal models were almost consistent with our prediction. Expression of β-catenin and vimentin was found to be higher in the tumor regions of mice with high CTSK expression while the expression of E-cadherin was low (Figs. 4A and S5A). Conversely, expression of β-catenin and vimentin almost disappeared in the tumor regions of mice with the negative CTSK expression while the expression of E-cadherin was high (Figs. 4B and S5B). Thus, whether CTSK could be a slicing machine for E-cadherin in PC cells was investigated next. CRPC cell line C4-2 was derived from LNCaP cell line [26, 27]. We found the primary C4-2 and LNCaP cells to resemble the epithelial-like cells, whereas C4-2-CTSK and LNCaP-CTSK cells resembled the mesenchymal-like cells (Fig. 4C). In order to identify the markers of EMT, we used the two cell lines for subsequent experiments. The protein expression levels (β-catenin, vimentin, snail and slug) in C4-2-CTSK and LNCaP-CTSK cells were enhanced compared to that in C4-2 and LNCaP cells. To test the function of IL-17A-CTSK-EMT axis, we introduced restructuring human IL-17A into C4-2 and LNCaP cells and found IL-17A to enhance the expression the CTSK in both of them (Figs. 4D and S5C, D). Interestingly, the enhanced IL-17A-CTSK expression had an effect on the protein expression of total E-cadherin, and the protein levels of slug, vimentin, twist, β-catenin, and snail were dramatically increased (Figs. 4E and S5E, F). Thus, we hypothesized that IL-17A influences EMT through CTSK, and that there is a strong correlation between the two. C4-2 and LNCaP cells were transfected with either CTSK siRNA or negative control siRNA, following which both of the cells were treated with restructuring IL-17A. Results showed that CTSK siRNA decreased the protein levels of CTSK expression induced by IL-17A. The protein levels of snail, β-catenin, E-cadherin, and slug were reduced in CTSK siRNA PC cells compared to that in the negative control siRNA PC cells (Figs. 4F and S5G, H). In brief, the results showed that CTSK protein level could increase by the induction of IL-17A and liberate β-catenin in PC cells. β-catenin can promote the release of transcription factors of EMT (snail, slug and twist), resulting in the enhanced expression of mesenchymal markers.

### Correlation of IL-17A/CTSK with the proportion of tumor-infiltrating immune cells in castration-resistant prostate cancer

**Fig. 5:**
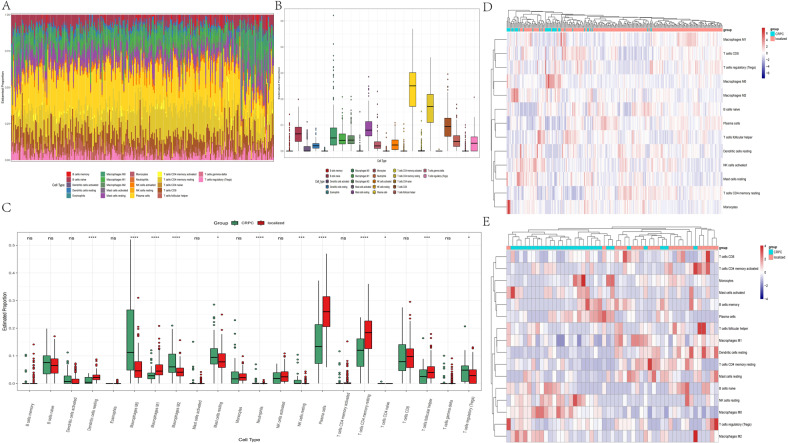
Immune infiltration in the combined sample dateset. **A** The bar chart shows the composition of the 21 immune-infiltrating cells at various sample, with each column representing a sample. **B** The histogram shows the composition of immune-infiltrating cells in all samples. **C** Comparison of the content of various immune-infiltrating cells in CRPC samples and localized PC samples. **D** The heatmap shows the amount of various immune-infiltrating cells in each sample, and each column represents a sample. The grouping of samples is shown in the annotation above. **E** The concentrations of various immune-infiltrating cells in the dataset for GSE32269 with each column representing one sample.

**Fig. 6:**
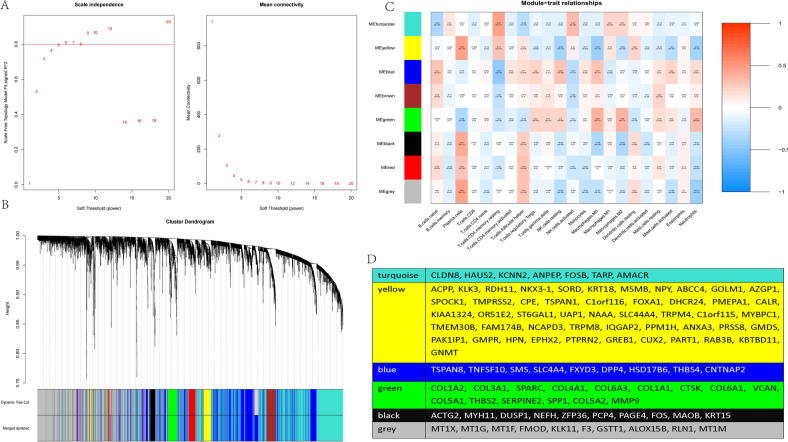
Correlation between the color modules and immune-infiltrating cells. **A** Determine soft-thresholding power in WGCNA. **B** Tree diagram of head 5000 genes gathered based on different metrics (1-TOM). **C** Correlation heat map between modules and immune-infiltrating cells. **D** The corresponding DEGs in each module.

**Fig. 7:**
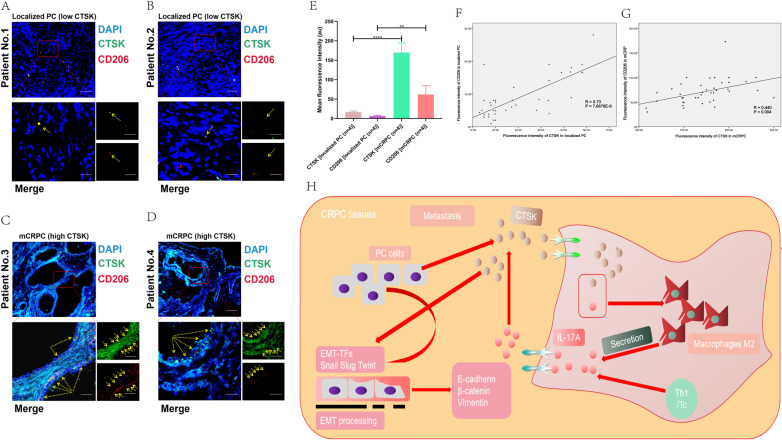
High expression of CTSK is related to high enrichment of M2 macrophages of tumor-associated in CRPC. **A**–**D** IF assay analysis of the correlation between CD206 and CTSK in prostate cancer tissues. **E** Quantification of immunofluorescence by confocal microscopy. Histograms show quantification of 10–25 different images per condition. **F** Correlation curve of CTSK and CD206 expression in localized PC tissues by quantification of immunofluorescence, eight sites are selected for each tissue. **G** Correlation curve of CTSK and CD206 expression in CRPC tissues by quantification of immunofluorescence, eight sites are selected for each tissue. **H** The profile mechanism of the regulation and mechanism of IL-17A/CTSK-mediated CRPC metastasis.

Using CIBERSORT algorithm, we analyzed the ratio of immune-cell subsets in tumor infiltration, and established 22 types of profiles of immune cells from PC samples (276 tumor samples with *p* < 0.05) (Fig. 5A) to demonstrate the relationship of immune microenvironment with IL-17A/CTSK. The histogram showed that the major immune-infiltrating cells associated with PC are T cells (T cells CD4 memory resting, T cells CD4 memory activated) (Fig. 5B). We, next, subdivided PC into the CRPC group and the localized PC group, and found the immune-infiltrating cells in CRPC to mainly be macrophages (M0 and M2), the infiltration degree of immune T cells was not as high as that in localized PC, indicating that macrophages have the most important influence on CRPC (Fig. 5C). The data were classified by heat map to check whether the infiltrating immune cells were mainly M2 macrophages in CRPC (Fig. 5D) and in the database GSE32269 obviously (Fig. 5E). All the genes were classified in order to clarify whether CTSK and M2 macrophages were correlated in CRPC. Before establishing the co-expression matrix withed weight, we defined the soft-threshold β as 6 in order to guarantee a scale-free topology. The degree of independence was 0.8 when the soft-threshold β reached 6 (Fig. 6A). Further, 5000 genes were confirmed in the GSE70770, GSE32982, and GSE32269 cohorts, in total, and the genes were classified into eight gene modules using WGCNA (Fig. 6B). Results showed the turquoise and green models to be strongly related to the M2 macrophages (Fig. 6C). Interestingly, IL-17A was in the turquoise model and CTSK was in the green model (Figs. 6C, D and S6). All the results indicated M2 macrophages to play a key role in CRPC, and possibly regulate immune microenvironment through the interaction of IL-17A with CTSK in CRPC. In the previous experiment, we had divided the nude mice into four groups based on the different expression of CTSK. The control + IL-17A group and the CTSK siRNA + IL-17A group showed higher expression of CTSK compared to the other two groups (Fig. 3N); therefore, we conducted flow cytometry on the tumor tissues of the four groups of nude mice to observe the immune cells infiltration. CD206 was found to be higher in the control + IL-17A and the CTSK siRNA + IL-17A groups than in the control and the CTSK siRNA groups (Fig. S7A). An immunofluorescence experiment was conducted next to further verify the relationship between M2 macrophages and CTSK. Results demonstrated that low expression of CTSK in localized PC is accompanied by low collection of M2 TAMs in stroma of the tumor (Fig. 7A, B, E). However, a higher expression of CTSK was seen in localized PC with the higher accumulation of M2 TAMs in stroma of the tumor (Fig. 7C–E). CTSK was often found to be strongly related to M2 in carcinoma of both localized PC and CRPC (Figs. 7F, G and S7B) when the correlation curves were used to further verify the relationship between CTSK and CD206 by immunofluorescence experiment and ELISA.

## Discussion

Patients with advanced-stage PC may develop metastatic castration-resistant prostate cancer (mCRPC) when they are administered ADT [28]. Owing to the fewer T cell infiltration in a tumor, mCRPC is prominently resistant to immune therapy. For example, an anti-PD-1/PD-L1 or anti-CTLA-4 single treatment of CRPC failed to exert a positive effect [29, 30]. Hence, understanding the progress of new immune-related nodes related to CRPC would be important.

Chemokines, such as IL-18A and EGFR, are released by M2 macrophages to enhance the invasion and migration of CRPC [31]. By checking chemokines which excreted by TAMs after CTSK therapy, some factors, like IL-10, IL-4, IL-8A, and their receptors, were found to be rapidly secreted due to the influence of CTSK [31]. On the other hand, the IL-17A receptor could stimulate NF-κB downstream molecules by mediating the formation of IL-17R-Act1-TRAF6 complex [32, 33]. The mentioned studies proved the key role of CTSK and IL-17A in the progression of CRPC.

In this study, a great deal of evidence was obtained that supported the influences of IL-17A/CTSK/EMT axis in the progression of PC. On one hand, the database was shifted to find the best evidence for the effect of CTSK on PC progression. On the other hand, CTSK was demonstrated as a gene related to metastasis, which could be up-regulated by IL-17A. The ability of invasion and migration of PC cells, enhanced by CTSK, was in common with the pivotal role of CTSK in different types of cancer. Moreover, CTSK promoted tumor growth in vivo and enhanced the ability of metastasis and invasiveness in vitro, showing its important function in tumor microenvironment and in improving the progress of PC. Therefore, CTSK inhibitors may be considered as a potential therapeutic targets to reduced tumor metastasis. Second, IL-17A could increase the expression of CTSK in PC cells and in tumor tissues of mice, and promote EMT marker expression through CTSK in PC cells. Finally, in the two PC cell lines, the ability of IL-17A-induced EMT was decreased when CTSK was knocked down. The result further proved that Il-17A induces the EMT process through CTSK.

In summary, IL-17A induced CTSK expression to disrupt E-cadherin/β-catenin complex, released β-catenin, and enhanced EMT and tumor cell invasion. Based on our study, IL-17A–CTSK–EMT axis was considered to be able to develop into a potential target for preventing and treating PC. The animal study demonstrated that CTSK siRNA reduced expression of EMT markers as well as the invasion ability of PC cells, further validating that IL-17A–CTSK–EMT axis is pivotal. Moreover, CTSK was shown as an important regulator in CRPC metastasis and could accelerate the progression of CRPC by promoting M2 polarization. Activated M2 macrophage could secrete chemokines to enhance the ability of metastasis and invasion in PC cells. Our study demonstrated the existence of a regenerative feedback loop that could affect the metastasis of CRPC (Fig. 7H), and proved CTSK as a potential therapeutic marker for CRPC.

## Supplementary information

Original western blots

Reproducibility checklist

Supplementary figures

## Data Availability

The datasets used and analyzed during the current study are available from the corresponding author on reasonable request.
