# Supplementary material for: Cathepsin K regulates the tumor growth and metastasis by IL-17/CTSK/EMT axis and mediates M2 macrophage polarization in castration-resistant prostate cancer
Source: Cell Death Dis. 2022 Sep 22;13(9):813. doi: 10.1038/s41419-022-05215-8 (PMC9499936; doi:10.1038/s41419-022-05215-8)

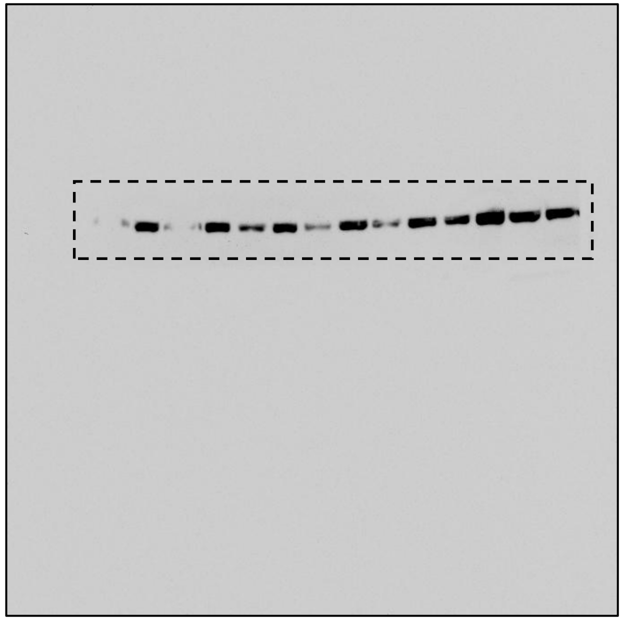

Figure 2A (CTSK) (N1-T7)

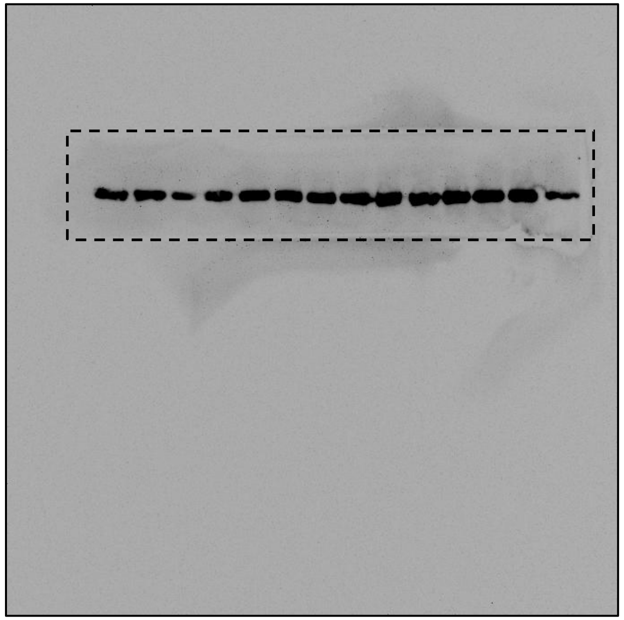

Figure 2A (GAPDH) (N1-T7)

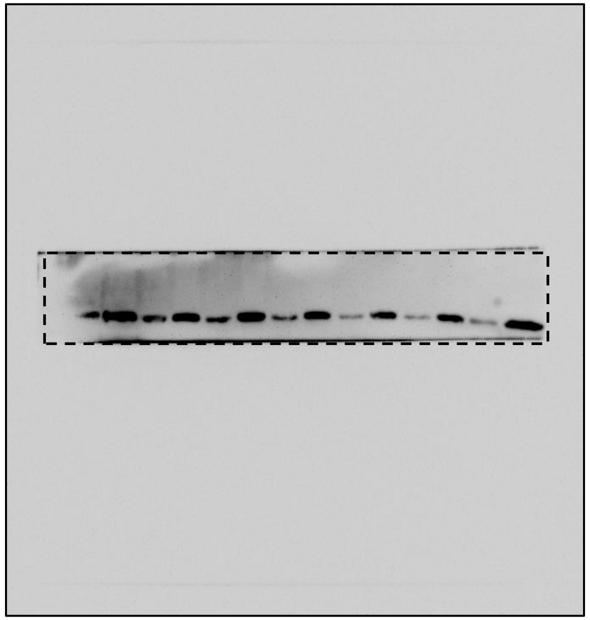

Figure 2A (CTSK) (N8-T14)

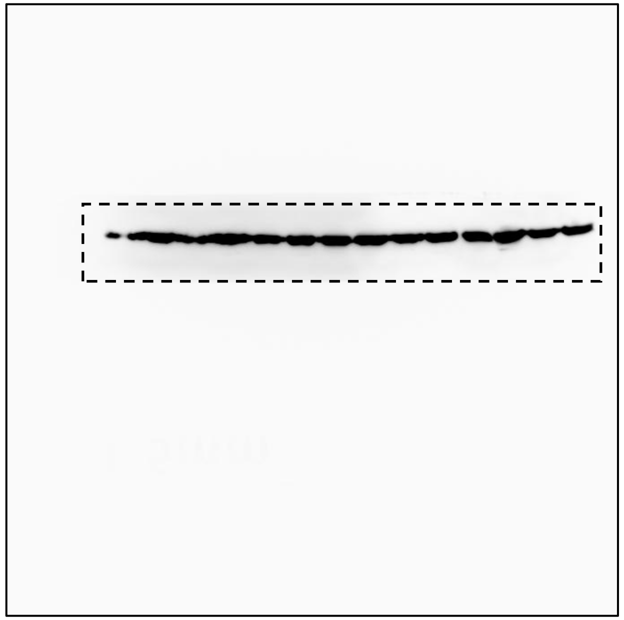

Figure 2A (GAPDH) (N8-T14)

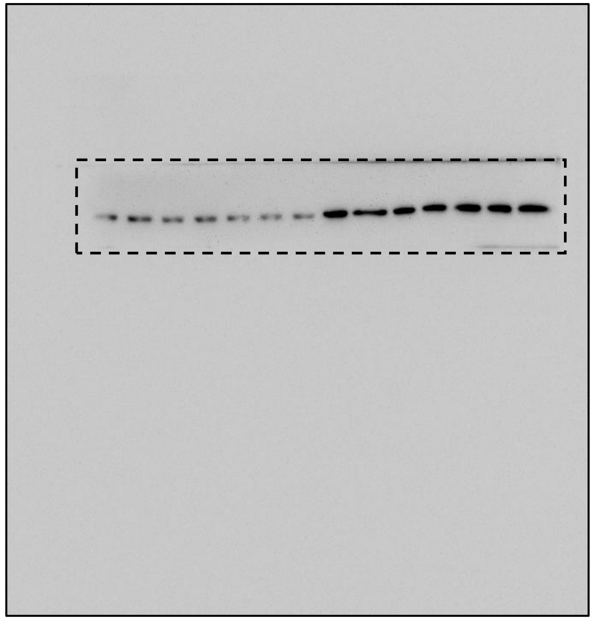

Figure 2C (CTSK)

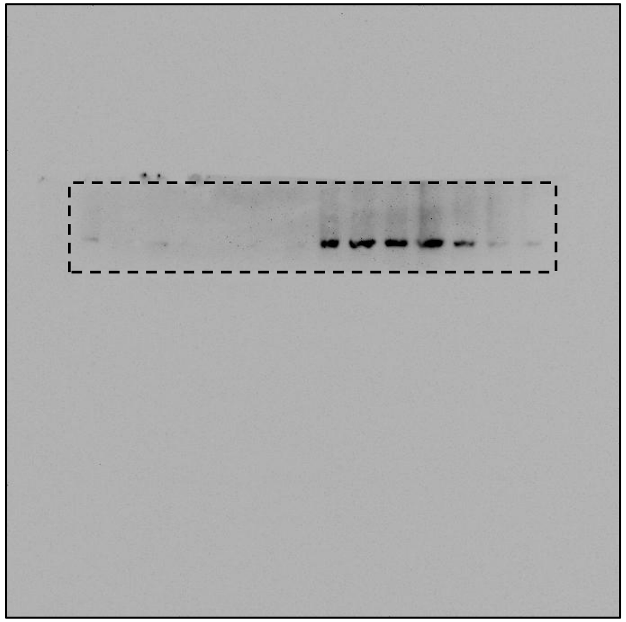

Figure 2C (AR-V7)

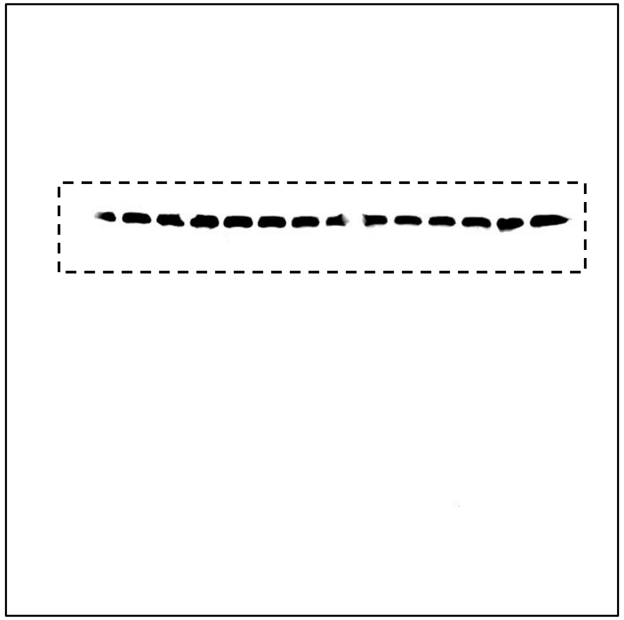

Figure 2C (β-tubulin)

Figure 3A (CTSK)

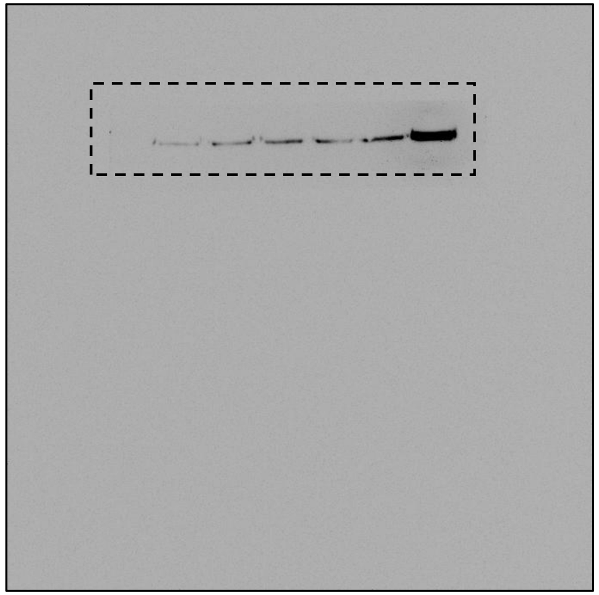

Figure 3A (β-tubulin)

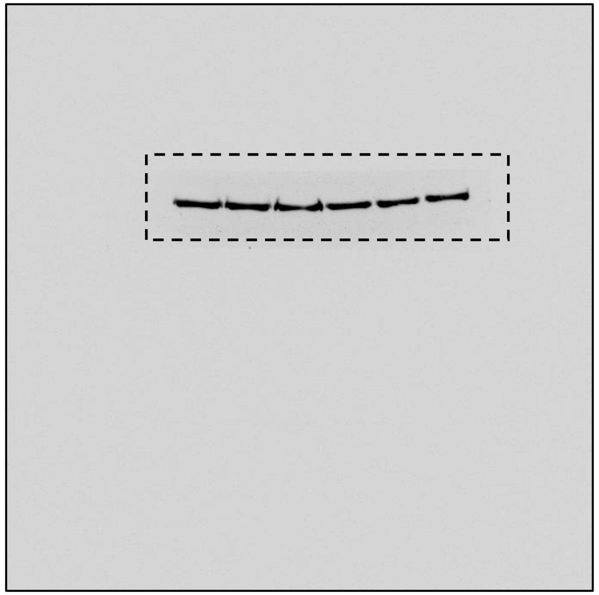

Figure 3C (CTSK)

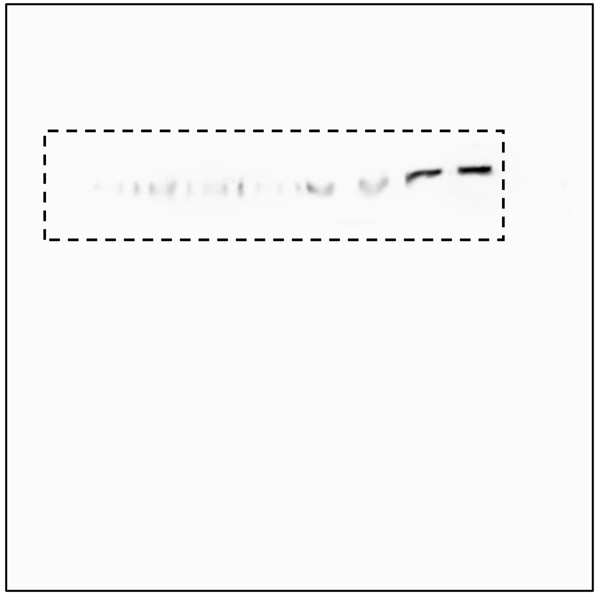

Figure 3C (GAPDH)

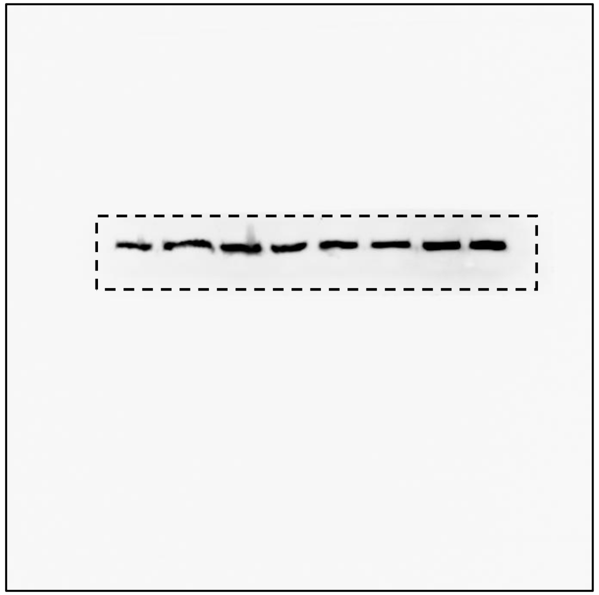

Figure 3B (CTSK)

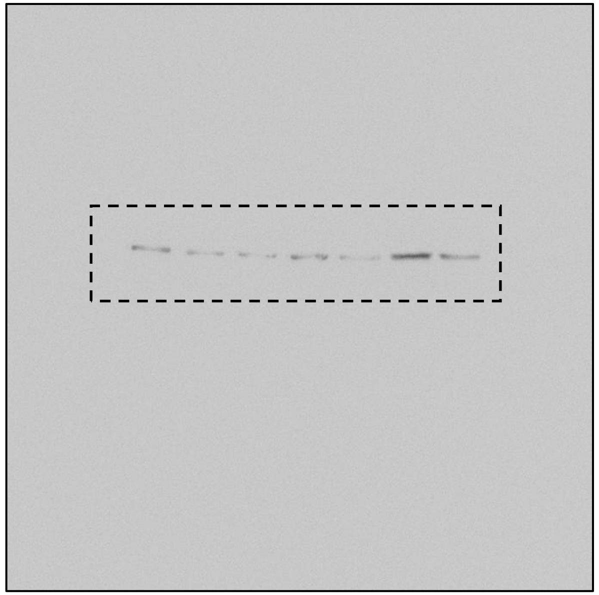

Figure 3B (GAPDH)

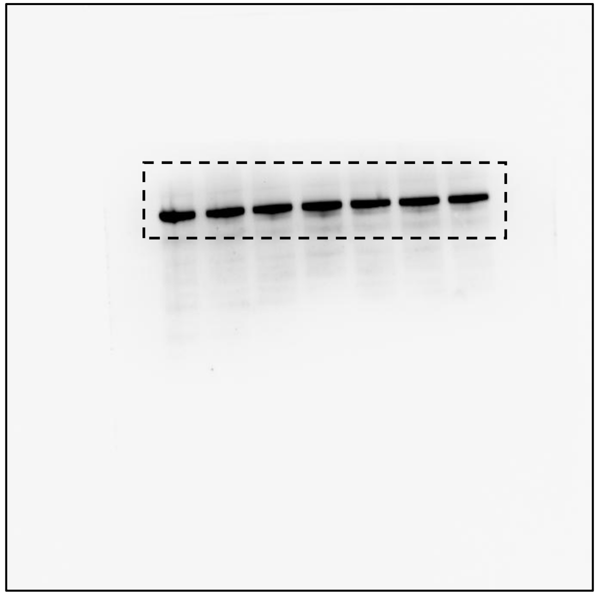

Figure 3C (IκBα)

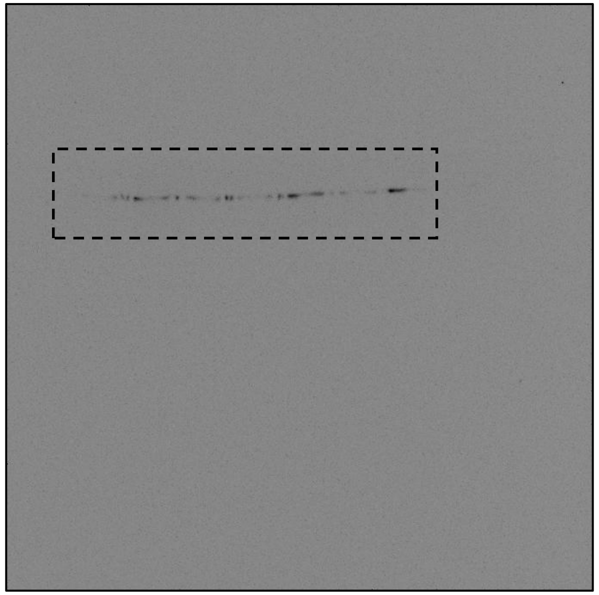

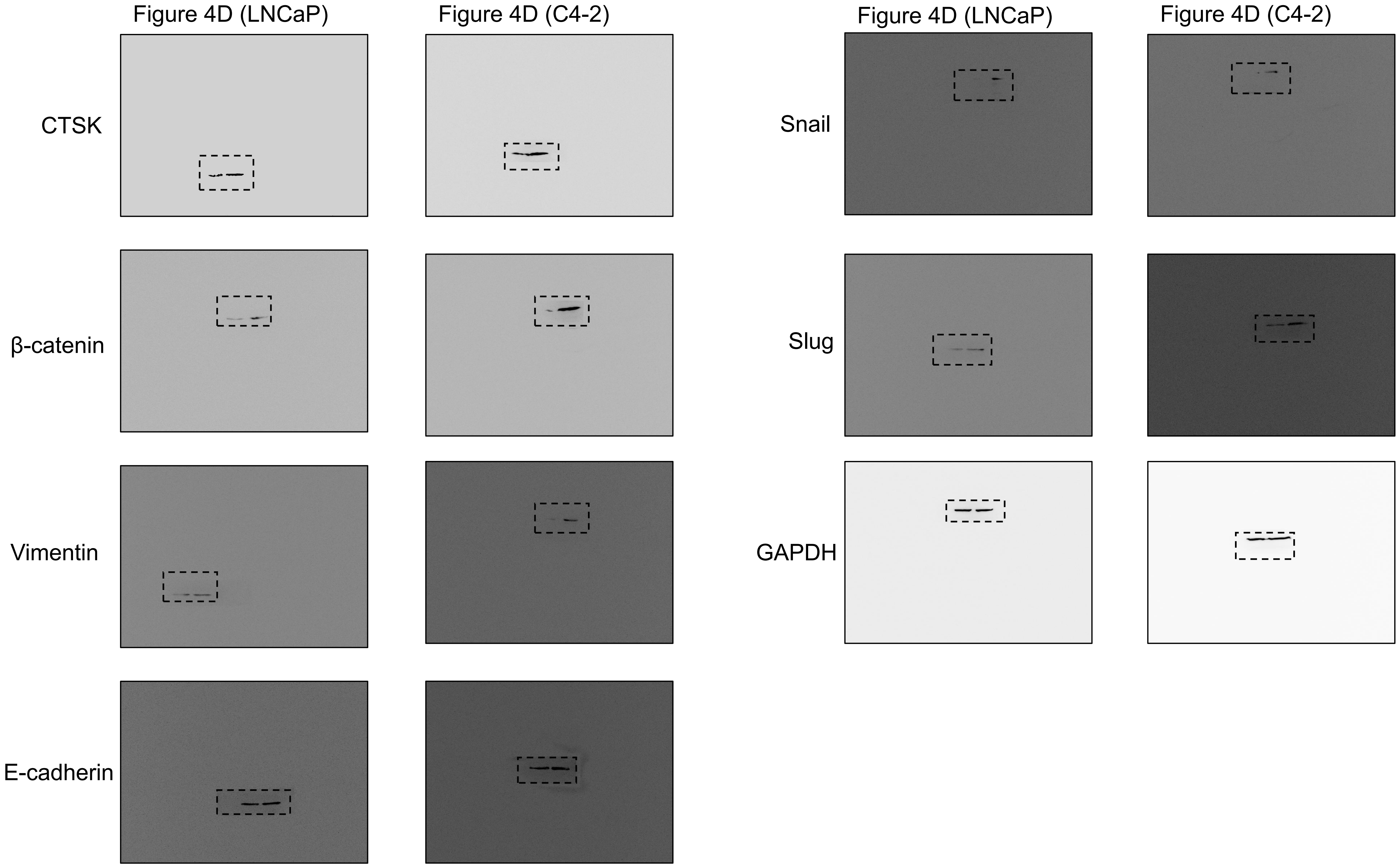
Figure 4D (LNCaP)
Figure 4D (C4-2)
Figure 4D (LNCaP)
Figure 4D (C4-2)
CTSK
β-catenin
Vimentin
E-cadherin
Snail
Slug
GAPDH

Figure 4E (LNCaP) (CTSK)

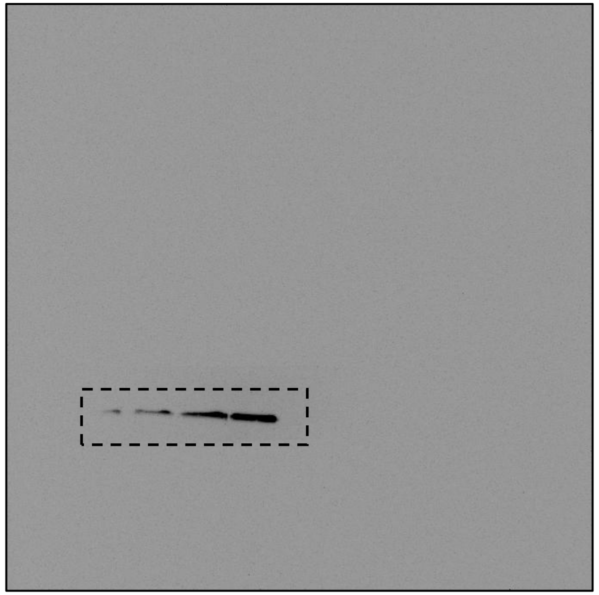

Figure 4E (C4-2) (CTSK)

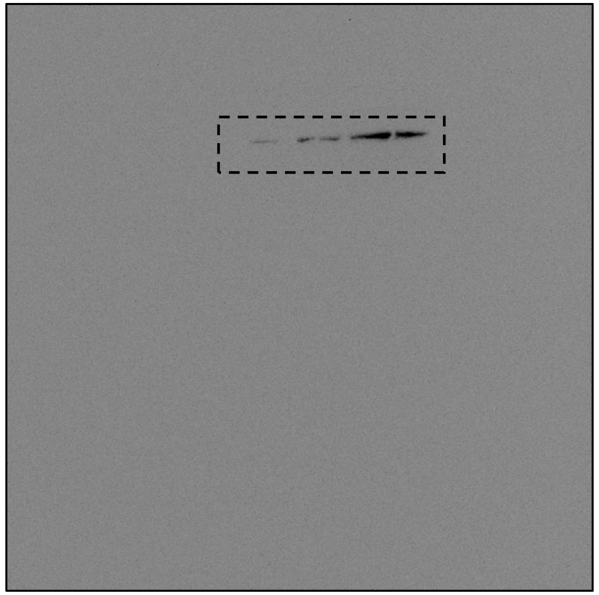

Figure 4E (LNCaP) (β-catenin)

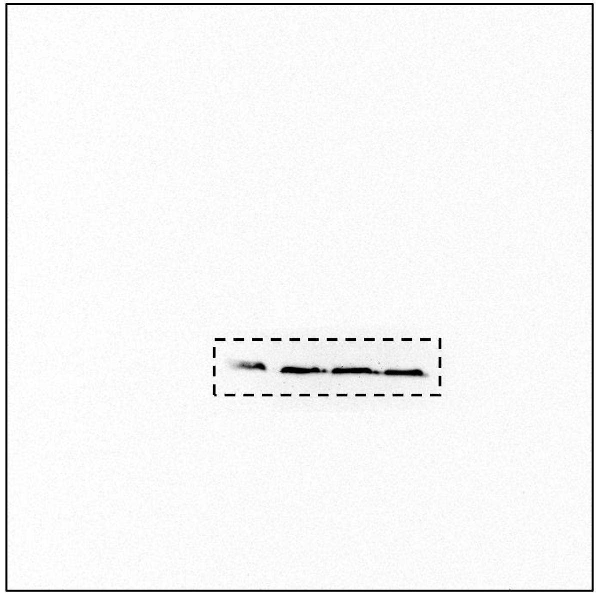

Figure 4E (C4-2) (β-catenin)

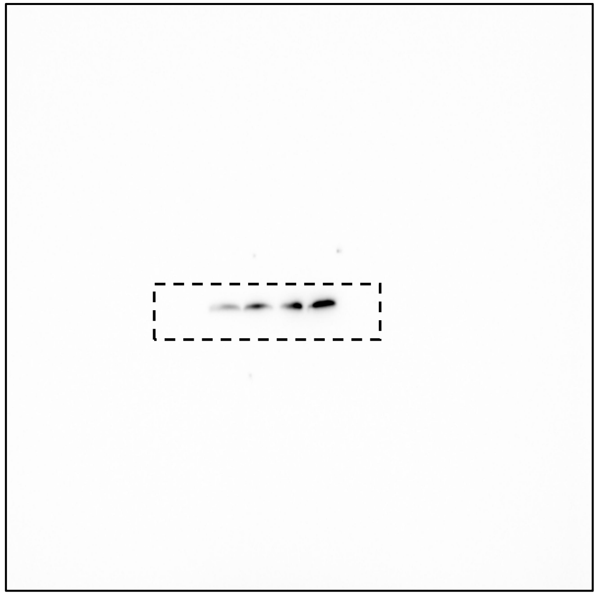

Figure 4E (LNCaP) (E-cadherin)

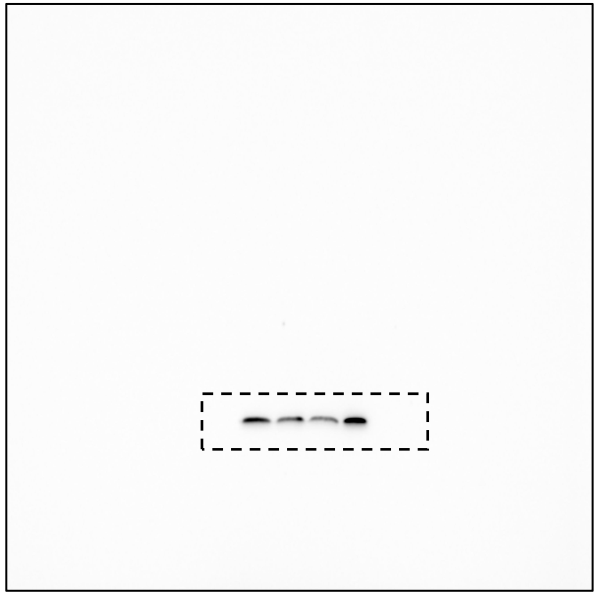

Figure 4E (C4-2) (E-cadherin)

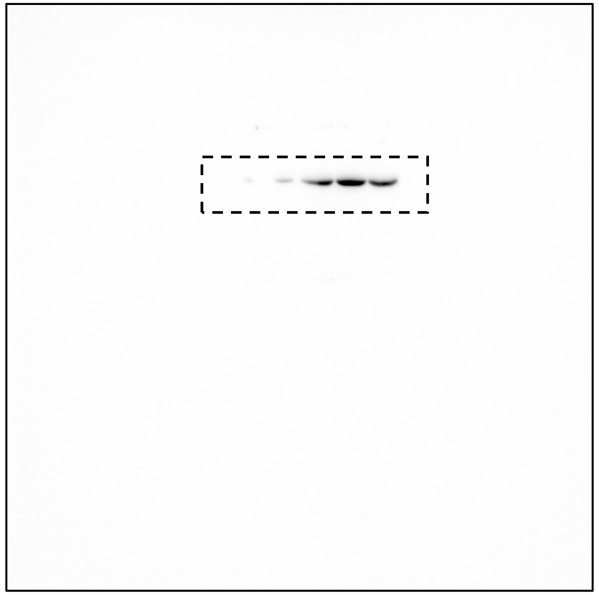

Figure 4E (LNCaP) (Snail)

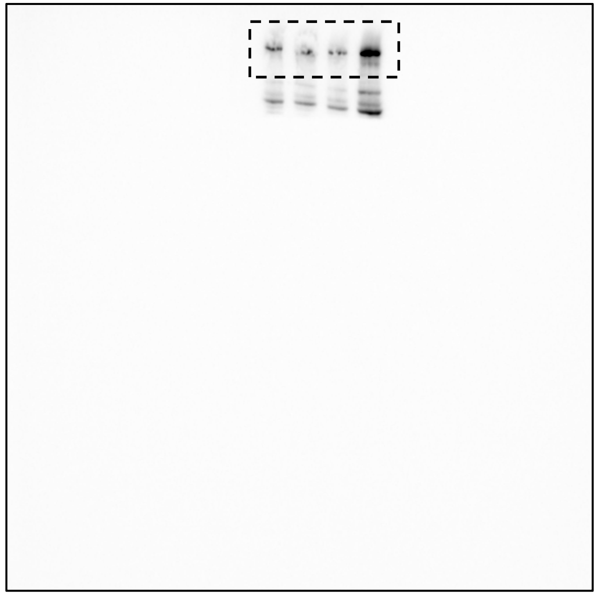

Figure 4E (C4-2) (Snail)

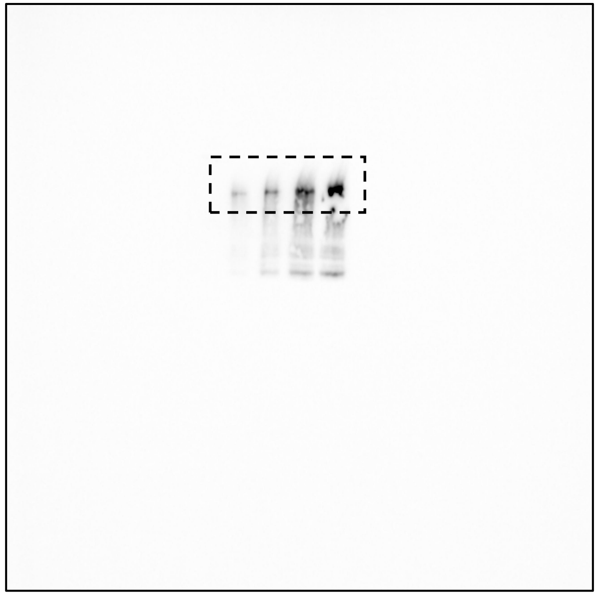

Figure 4E (LNCaP) (Slug)

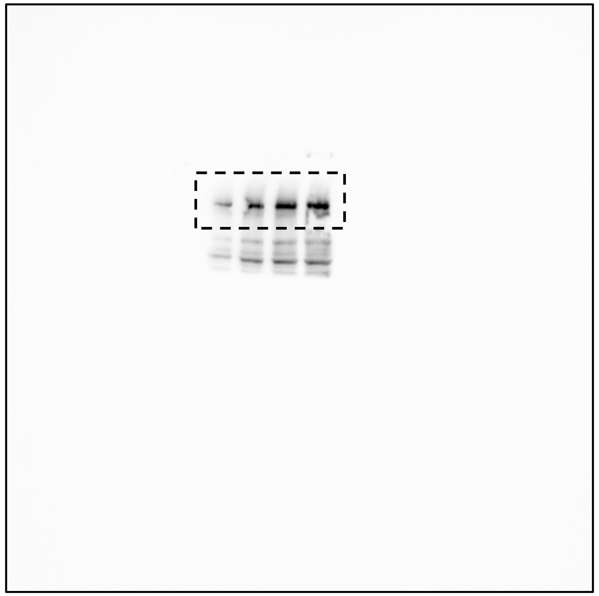

Figure 4E (C4-2) (Slug)

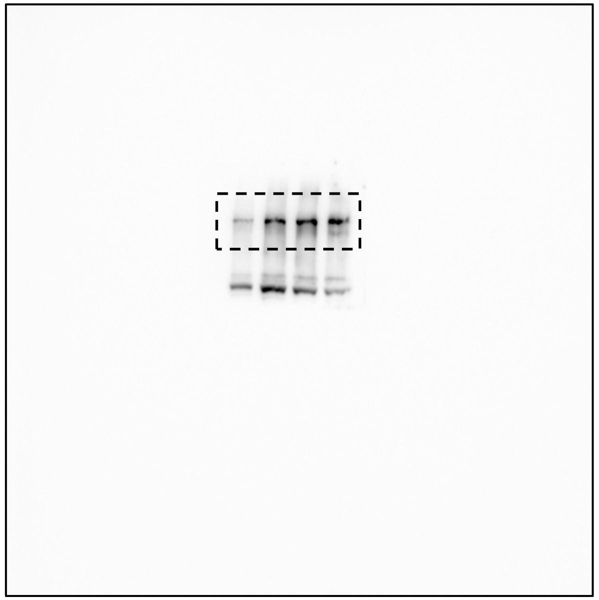

Figure 4E (LNCaP) (Twist)

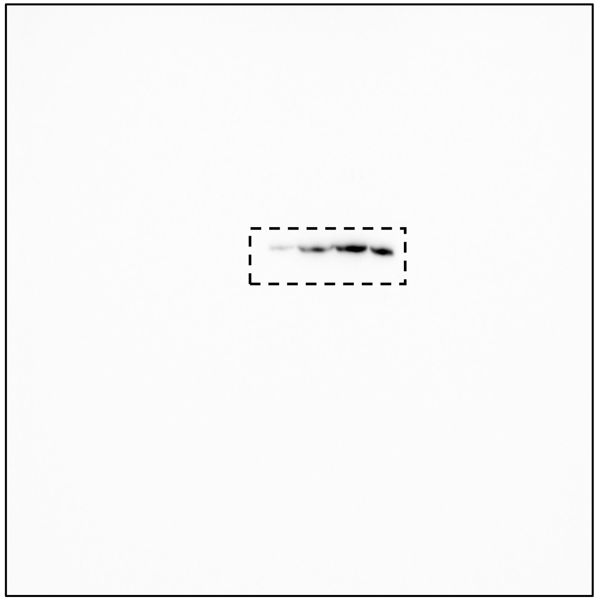

Figure 4E (C4-2) (Twist)

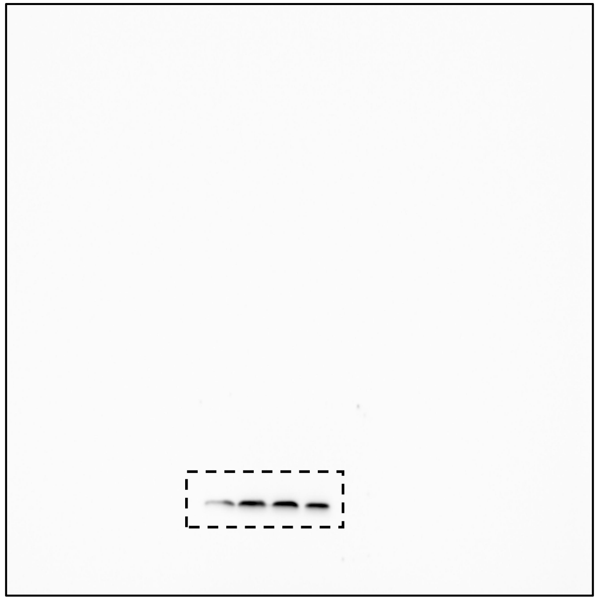

Figure 4E (LNCaP) (GAPDH)

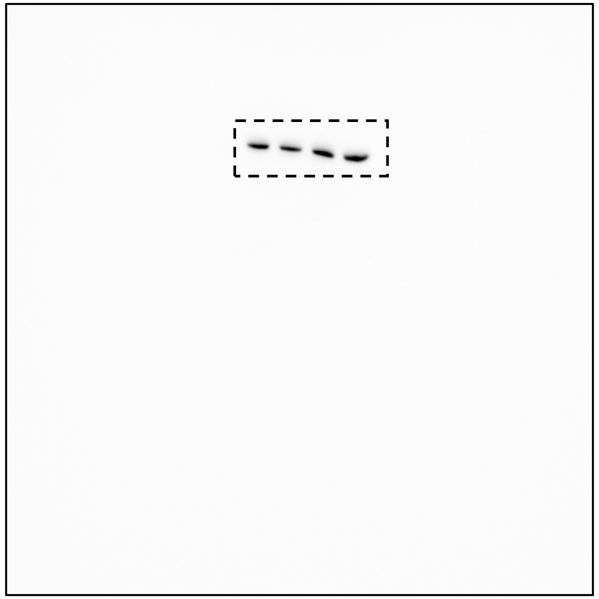

Figure 4E (C4-2) (GAPDH)

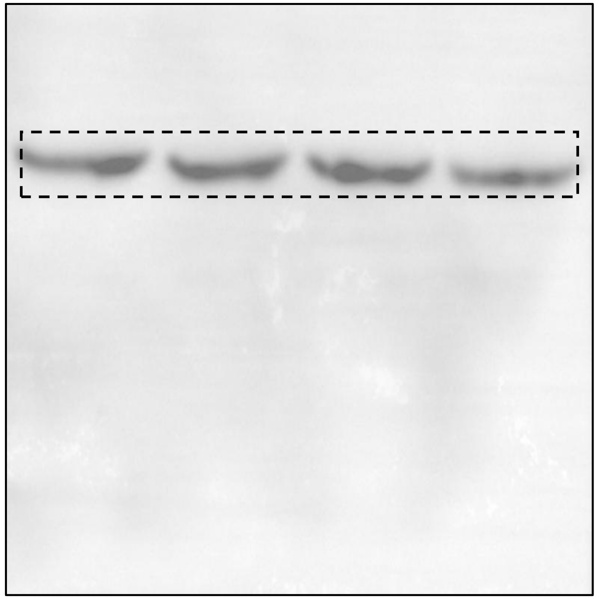

Figure 4F (LNCaP) (CTSK)

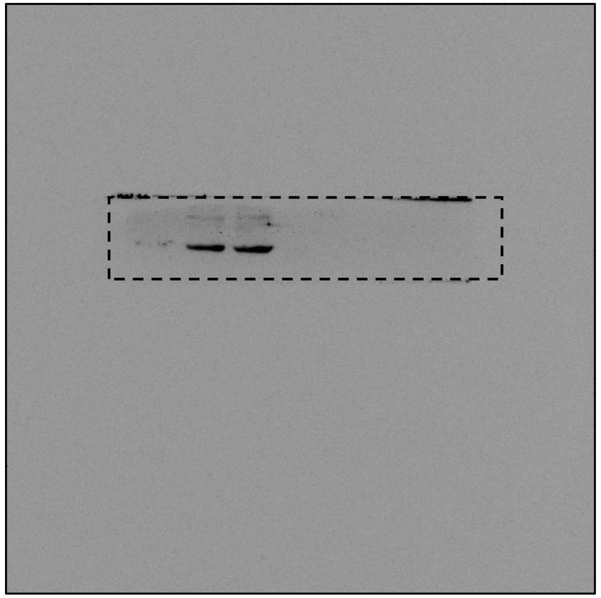

Figure 4F (C4-2) (CTSK)

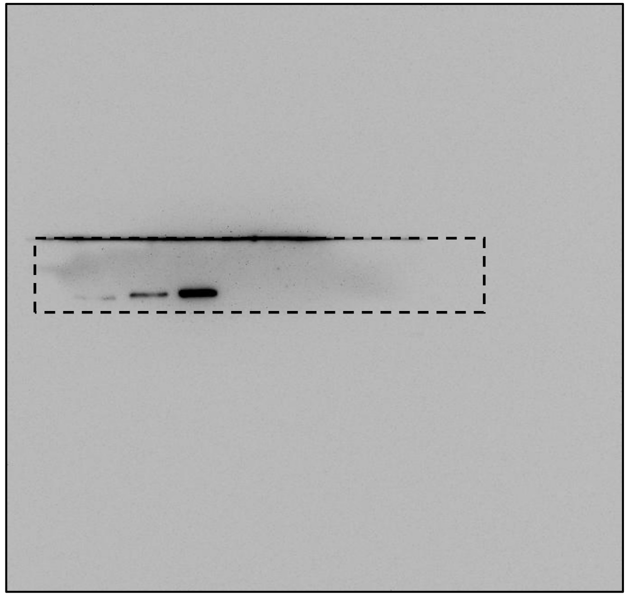

Figure 4F (LNCaP) (β-catenin)

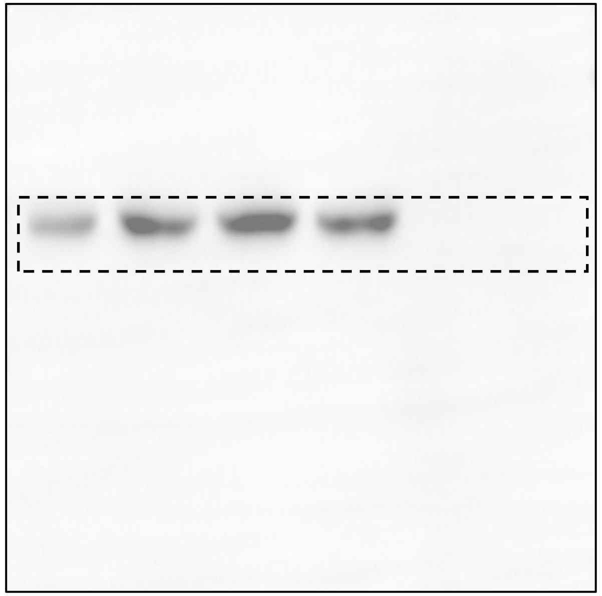

Figure 4F (C4-2) (β-catenin)

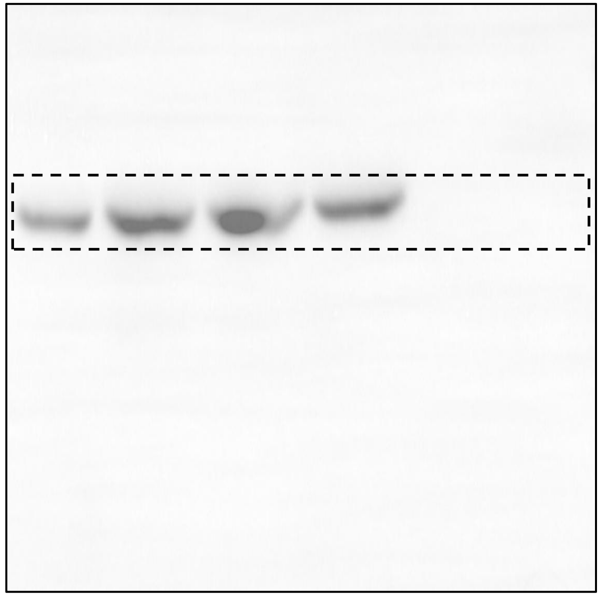

Figure 4F (LNCaP) (E-cadherin)

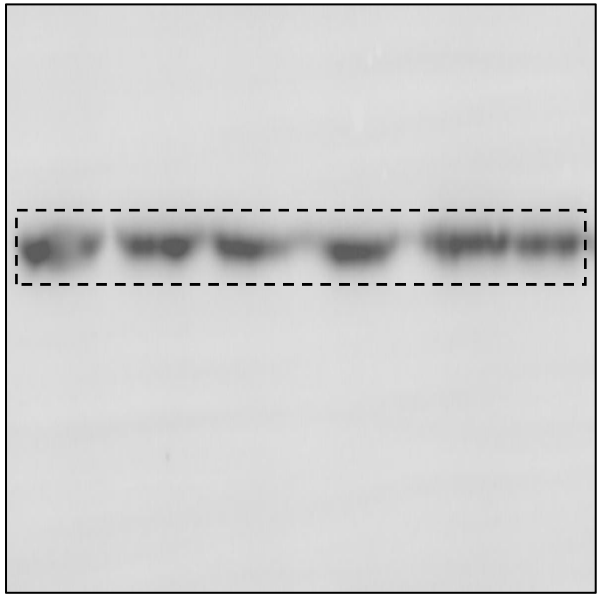

Figure 4F (C4-2) (E-cadherin)

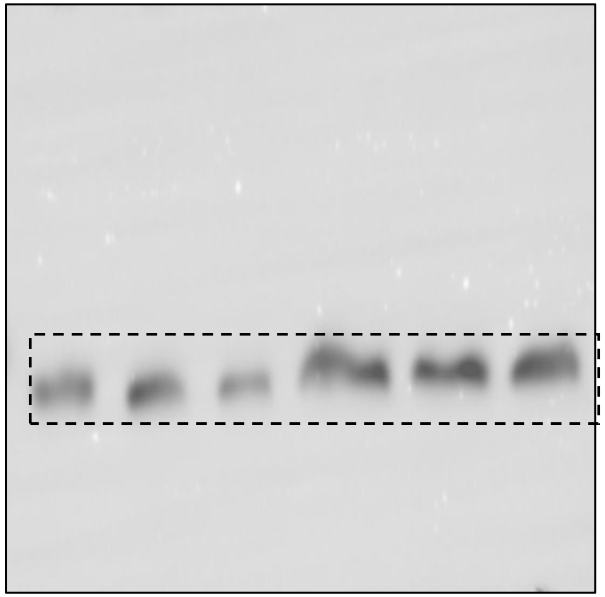

Figure 4F (LNCaP) (Snail)

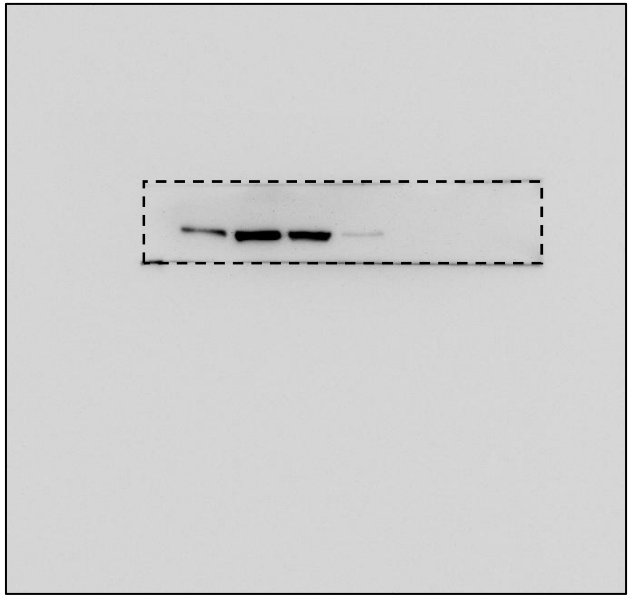

Figure 4F (C4-2) (Snail)

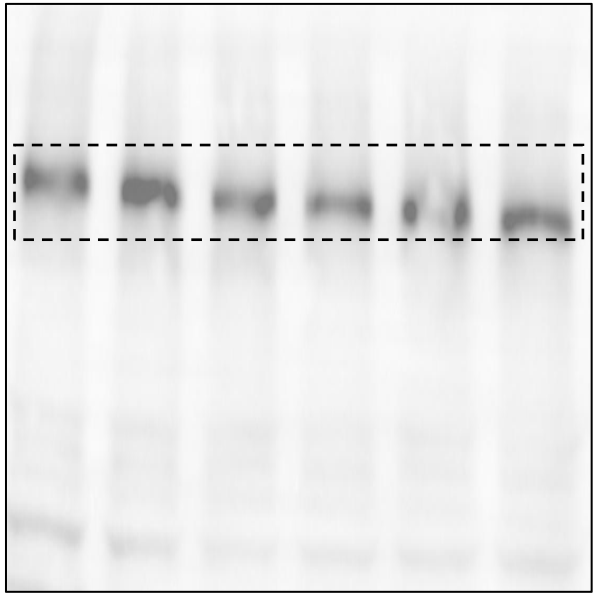

Figure 4F (LNCaP) (Slug)

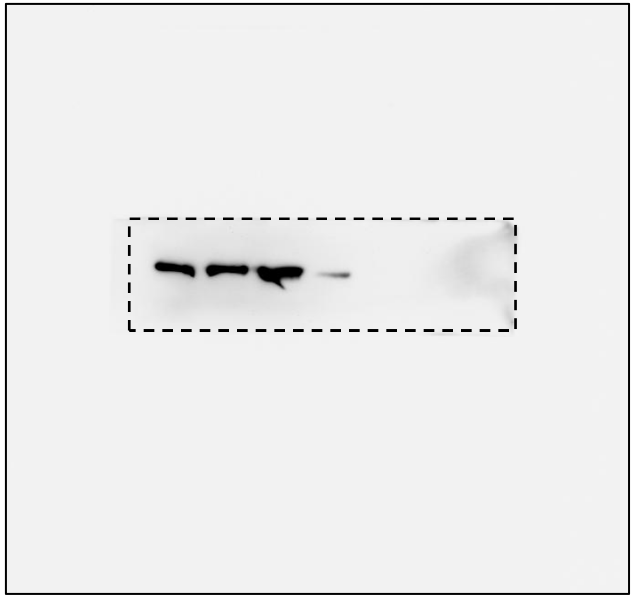

Figure 4F (C4-2) (Slug)

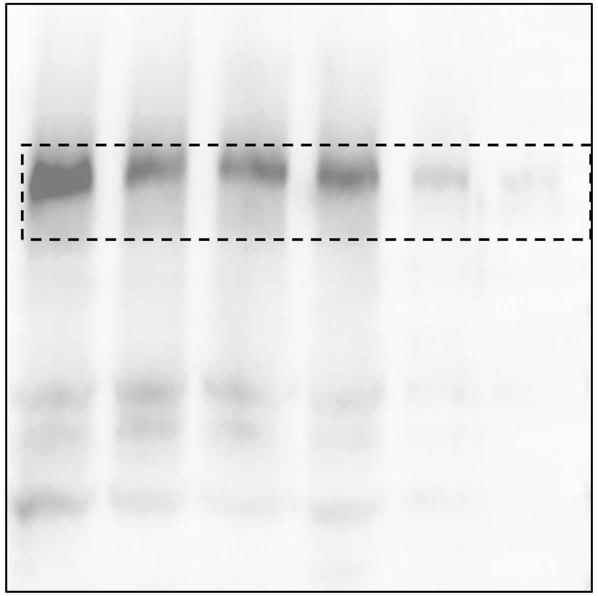

Figure 4F (LNCaP) (GAPDH)

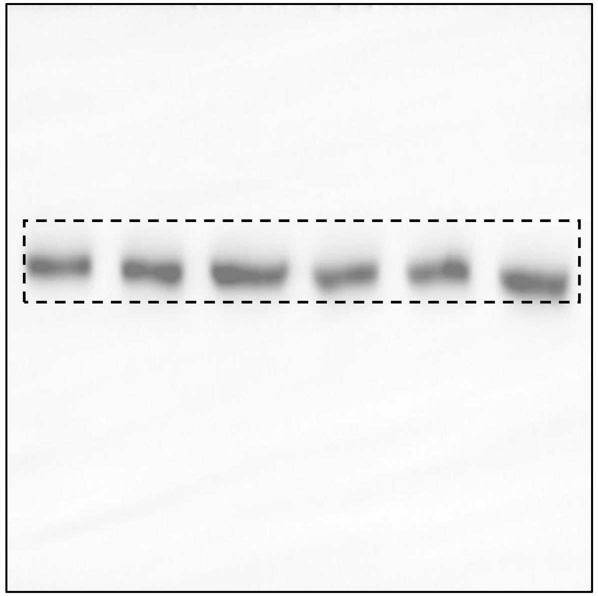

Figure 4F (C4-2) (GAPDH)

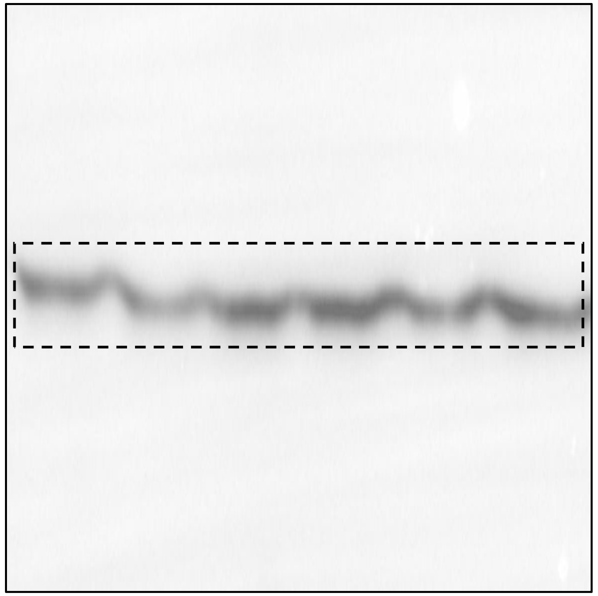

Supplement: Supplementary file 1 — Original western blots [file 41419_2022_5215_MOESM1_ESM.pdf]
