# Supplementary material for: Cathepsin K regulates the tumor growth and metastasis by IL-17/CTSK/EMT axis and mediates M2 macrophage polarization in castration-resistant prostate cancer
Source: Cell Death Dis. 2022 Sep 22;13(9):813. doi: 10.1038/s41419-022-05215-8 (PMC9499936; doi:10.1038/s41419-022-05215-8)
Supplement: Supplementary file 3 — Supplementary figures [file 41419_2022_5215_MOESM3_ESM.pdf]

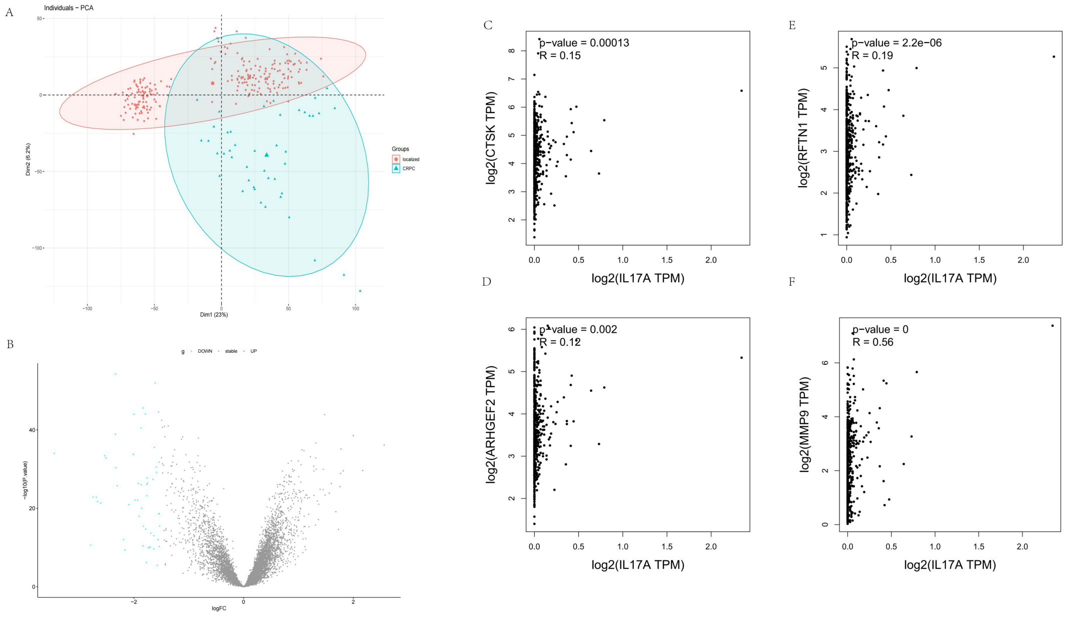

Supplementary Figure 1

(A) Two-dimensional plots of localized PC and CRPC group with the top two principal components of three separated datasets after correction. (B) Heatmap for top 105 genes differentially expressed in localized PC and CRPC in GSE70770，GSE32982 and GSE32269. (C-F) The relationship of IL-17A and CTSK, RFTN1, ARHGEF2, MMP9 in GEPIA.

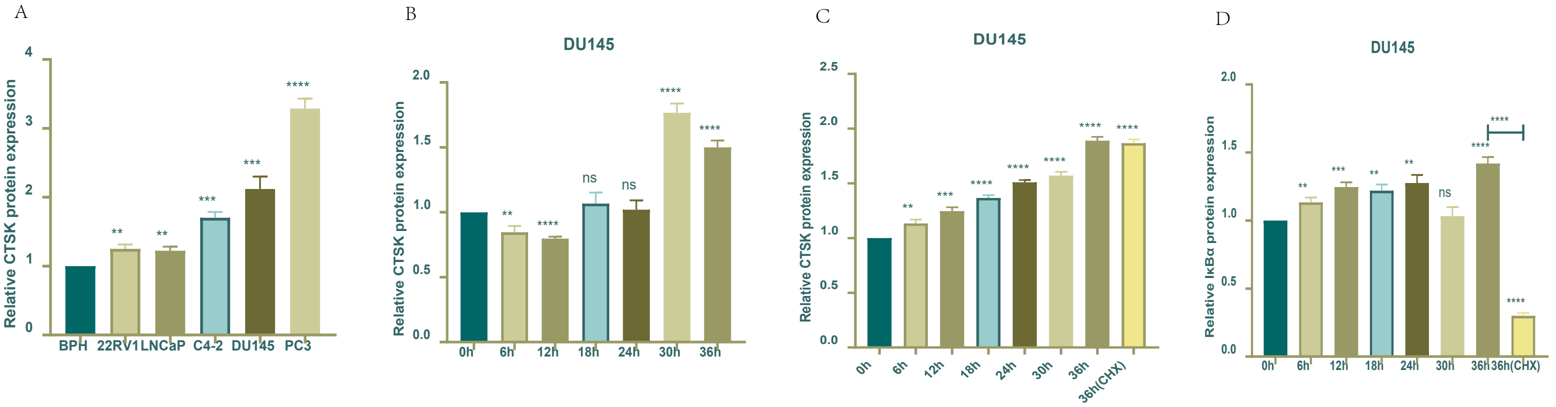

Supplementary Figure 2

(A) Quantization of Figure 3A. (B) Quantization of Figure 3B. (C-D) Quantization of Figure 3C .

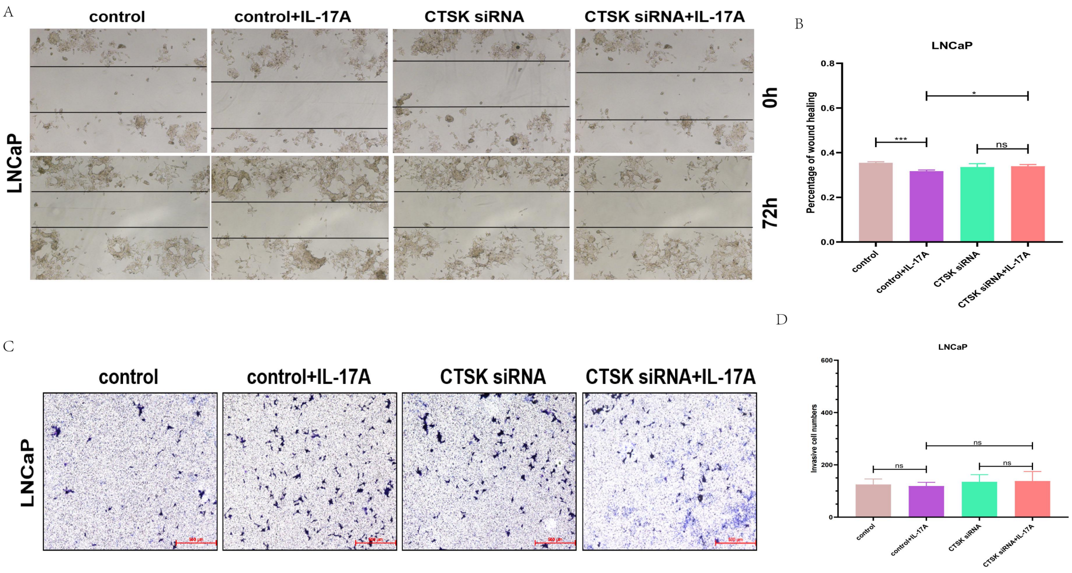

Supplementary Figure 3

(A) Wound healing assay in LNCaP cells transfected with negative control siRNA, IL-17A, CTSK siRNA, CTSK siRNA+IL-17A. (B) Quantization of A. (C) Transwell assay in LNCaP cells transfected with negative control siRNA, IL-17A, CTSK siRNA, CTSK siRNA+IL-17A. (D) Quantization of C.

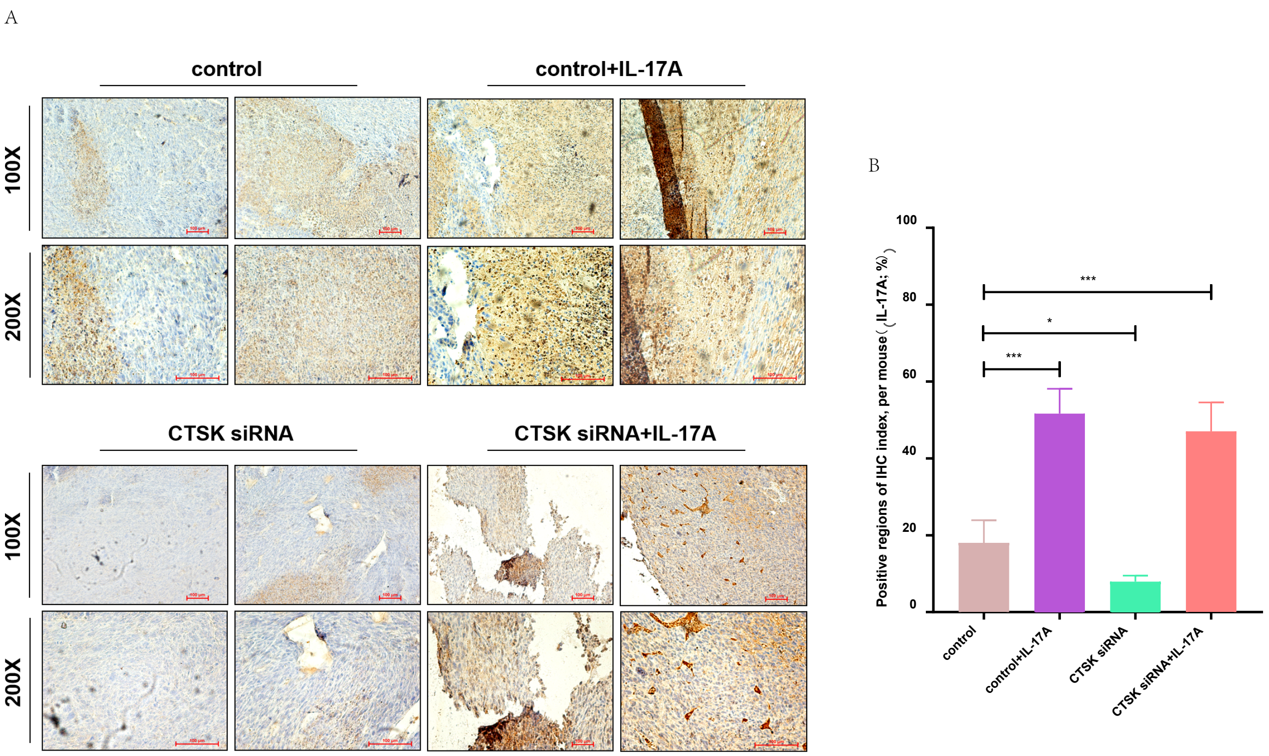

Supplementary Figure 4

(A) Comparing C**TSK** expression in negative control si**RNA, IL-17**A, C**TSK** si**RNA**, C**TSK** si**RNA**+**IL-17**A groups by

IHC staining. (B) **Quantization of A.**

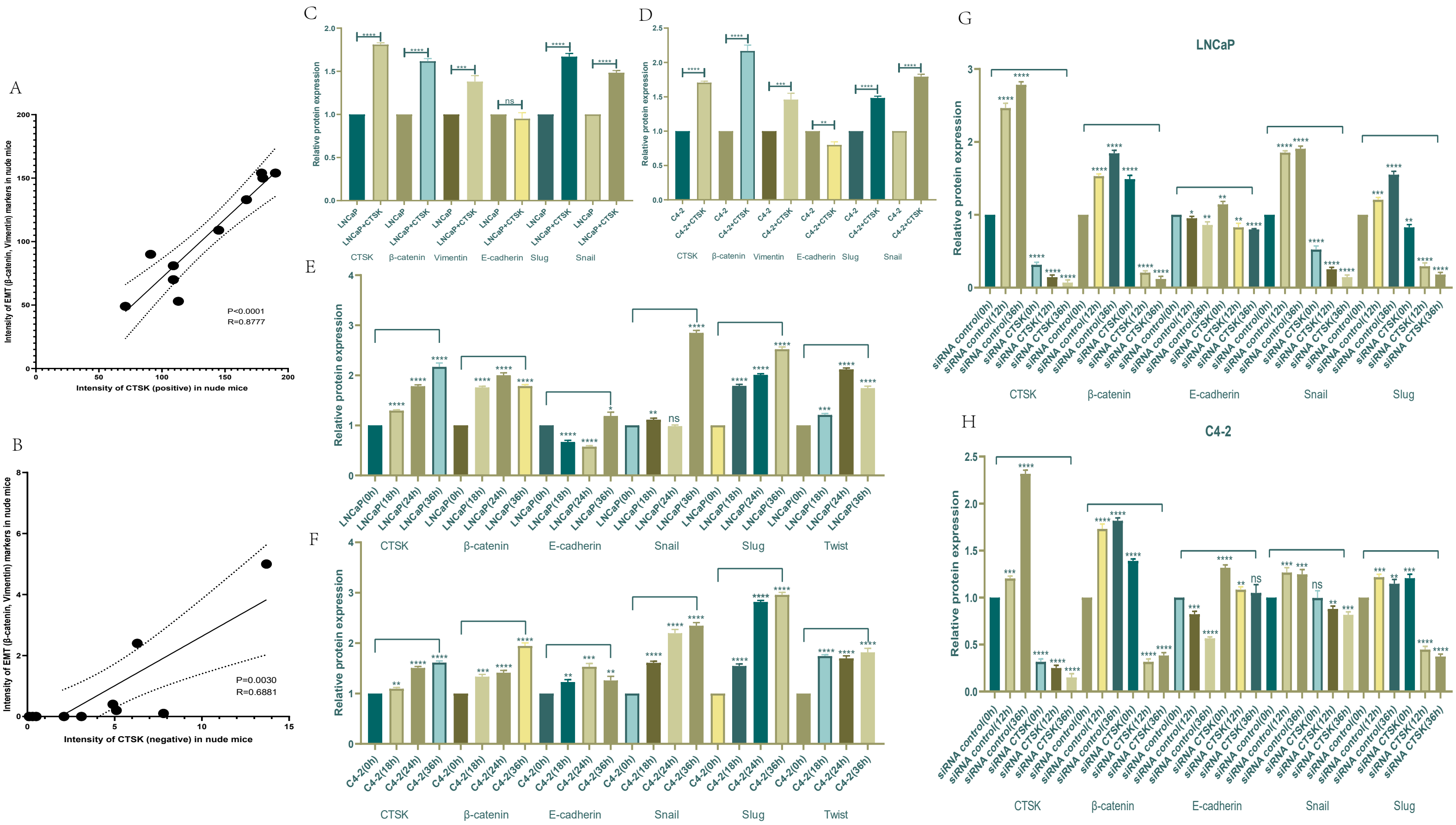

Supplementary Figure 5

(A-B) Comparing the correlation between CTSK and EMT markers in different nude mice groups. (C-D) Quantization of Figure 4D. (E-F) Quantization of Figure 4E . (G-H) Quantization of Figure 4F.

turquoise	RPS2 RPL23A RPL31 RPS27 RPLP0 TPT1RPS16 RPS18 RPS6<br>RPL9RPS14 RPS12 RPL32 RPL15 RPL30 RPS3 RPL24<br>RPS29 RPL12 B2M RPL27 RPL38 RPS17 FTL RPS15A RPL18<br>ACTB RPL22 UBB RPL19 RPL8RPS25 RPLP1 RPS5 RPL29<br>RPL14 RPL3RPL35A FAU SAT1EIF3L TMA7 YWHAQ BTF3<br>ATP5F1A LAMP1 EIF1 HSP90AA1 ATP5F1B RPL35 RPL36 NCOA4<br>HSP90AB1 SOD1 COX7C MYL12B GLO1 ITM2B RPL41<br>HMGN2 RPS21 SRP14 ARF4 COX7A2 PSMB1 TMEM258<br>SLC25A3 TUBA1B PPIA SRP9 RPL34 EEF1D HSPD1 SKP1<br>HLA-A EIF4G2 UQCRQ COX6C NPM1 SLC25A5 DYNLL1 SEC61B<br>PLPP1 NDUFA4 ATP5PB SELENOP DAZAP2 YBX1 COPB1 TMED2<br>BEX3 CHCHD2 MAGED1 REXO2 COX5B SLC39A6 TBCA H2BC12<br>ANXA5 ATP5F1C ALDH9A1 CNBP MORF4L2TRAM1 YWHAZ<br>ATP6V0E1 EIF4B ODC1 SET TSPAN13 MPC2 MORF4L1<br>ATP5MC2RAB11A IDH1CD9 EIF3H SEC11A EIF3F VDAC3<br>HSD17B4 SNX3 PHB2 PSMB4 NFIB COX6A1 ATP5PO CCT8<br>ATP5PF SPCS1 PSMC1 TAX1BP1 ATP5PD FOXO3 ATP6AP2 PSMA1<br>HDAC1 BTG1 TALDO1 ZMYND11 POMP CXADR ATP6AP1<br>MARCKSL1 DAD1 NSA2 DEGS1 NDUFA1 PGK1 AHNAK<br>TMBIM4 HNRNPD EIF3K TMEM123 ERH EIF4H HMGN1<br>UQCRFS1 DNAJA1 PRKAR1A COX5A SNRPG H2AZ1 SERP1 PPP3CA<br>RHEB STEAP1 TNPO1 SSR2CANX MDH1 GOLPH3 ESD XRCC6<br>APEX1 CCT2 ATRAID PTTG1IP SF3B1 PGRMC1 NFKBIA CYB5A<br>MRPL3 DYNLT1 TOMM20 TMED10 EIF3IHIGD1A TXN SSR1VDAC2<br>TPI1 NAP1L1 SEC61G RAB13 PAPSS1 VPS35 MGST2 UQCRB<br>ARL6IP1 TRMT112 GHITM RTRAF HNRNPK ECHDC2 STARD7 TMCO1<br>SUB1 PTGES3 PSMC2 DYNLRB1 PACSIN2 CSTB PRDX2<br>GABARAPL2 KDELR2 GTF3A DHX15 TSC22D3 MRPL33 YY1<br>ATP6V1A HSPB1 PCBP1 UBE2N ATP6V0C PFN2 MCL1 DCN<br>DYNC1LI2ANXA7 HSD17B12 ATP5MPL LAMTOR5 PTBP1 FNTA<br>ANAPC5 CSNK1A1 CTTN YIPF6 MGST3 NME1 UXT POLR1D<br>ZMPSTE24 BZW1 LTA4H NPTN CCT3 RAB3GAP1 AARS1<br>CPNE3 PDHB EIF4A3 SNRPB NOL7 NHP2 PUM1<br>HNRNPA1CAMLG EIF3B RNF103 CAB39 RAB7A SLIRP ARPC5<br>AZIN1 PSMB5 MAPRE1 PSMA4 EFR3A EZR RRAGA GLG1<br>TMX2 PSMA5 WDR6 UQCRC2 SUCLG1 NDUFA3 RNF7 SRSF3<br>CCND1 CAT ADD1 STRAP PFN1 CYCSACAT1 HNRNPC SHC1<br>PDIA6 CREG1 H2AC6 UGDH MICU2 FKBP1A SYPL1 TPD52<br>C6orf62 SMARCC1 PIGP C5orf15 PGAM1 MARCHF6 PSMB2<br>APMAP TSPYL1 DAP3 TJP1 DECR1 CHD9 ARL8B PPP2CB<br>MRPL20 LMBRD1 ZBTB20 UQCR10 NUCB1 NDUFS3 PCBP2 RGS10<br>USO1 MACROH2A1 JPT2 NISCH PPA2 PDCD10 NET1 DUT<br>SRSF7 MKNK2 PMPCB RPN1 GNG5 CLNS1A NUCKS1 CYB5R3<br>GTF2H5 URI1CSE1L CDIPT GGCT CCT4 GLRX5 ECI2

	GOLGA7 TSPAN3 MRPL24 PLXNB2 DUSP3 HAX1 TSG101 IARS1<br>SLC12A2 CMC2 ANXA11 CEBPB GSPT1 POLB NDUFB6<br>TMEM147 DDX21 ADSL SAR1B RING1 NIPA2 CNIH1<br>WDR61 TMEM14B NSF CGGBP1 STAG2 TOR1AIP1 SNRPB2<br>KIAA0355 COMMD3 CAPZB FBXO21 NDUFC1 CCNG2 G3BP2<br>ENSA WDR45B PSMD7 TM2D3 SEC23B CLCN3 MMUT NFE2L1<br>UBE2L3 LYPLA1 MRPS33 ACTR10 EPRS1 DHFR NUDT9 TMED5<br>RAB2A DEK MRPL18 VBP1 CNOT7 UQCR11 AK2 ADAM10 RIOK3<br>PWP1 TAF7 TUT7 HIF1A UNC50 DPM1 ENOPH1 DCAF6<br>BLCAP AP3S1 HNRNPA0 IER2 USP7 EI24 DDX18 SPTBN1 COPS5<br>CPD SIAH1 MTMR12 SEC24C PSMD14 IARS2 CHPT1 THAP12<br>HDAC2 RBL2 SUMO3 CALU CDV3 PEA15 BRD3 LIPA<br>COLEC12 PTDSS1 PDCD6 PARM1 SMARCA2 LSM5 LANCL1<br>PSMD6 ARMCX6 RUSC1 ZNF22 FLOT2 SRP54 HMG20B BNIP3L<br>METAP1 KRT10 HSD17B10 RALB SDHD YME1L1 PRKCSH<br>EIF1B CDK4 GNPAT PUM2 HMGN4 LRPPRC BSG ARHGAP1<br>ILF2 SLC30A9 CTDSP1 AIMP2 BZW2 CHMP5 YTHDF3 RAD21<br>CLINT1 LAP3 TRIM33 GGPS1 PLA2G7 STOML2 NFE2L2 FH<br>SRP72 ARHGEF7 PSMC6 TSEN34 COASY RTL8C SNRPA1 EWSR1<br>PON2 PHKB LGMN DYNLT3 VPS4B VWF UBE2G1 SMIM14<br>HNRNPU TRIM8 NIPSNAP2 SPSB3 ACP1 SEPHS2 EIF2AK2<br>HSD17B11 RAD23B RARS1 PA2G4 LSM3 HACD3 PRPF19 SRI<br>LIMA1 MARCHF7 LAMTOR3 VAMP7 ETFA TRAPPC8 GLT8D1<br>MYO6 MAPK6 YARS1 ORC5 FAM50A EIF2D ARHGDIA HARS1<br>SYNCRIP TAPBP SPCS3 DLD TMEM126B ETFB ACBD3 C1QBP<br>TBL1X YTHDF2 TARS1 SSB HLTF PSME1 DYRK1A MCFD2 HIBCH<br>KPNA6 MAGT1 BUD23 PRRC1 SPG21 METTL3 GTF3C1 MLH1<br>FNBP1L PCBD1 RXYLT1 PTS VEZF1 EVL PSMD10 PLEKHB2 ZFAND6<br>CBY1 GALNT1 ATP6V1D PNPLA2 SLC35B1 ITFG1 ADNP DCTPP1<br>CDKN1B GTF2A2 ARF3 CLDN8 HSD17B7 SRSF8 TCF7L2<br>TMEM248 LSM7 CES2 GNAI3 MRPL49 NOL11 OFD1 ELOC<br>RPL26L1 SPTLC1 NAE1 PCCB PIK3R1 ASH2L H2AW MYC<br>RAB9A SASH1 BAG3 DPH5 RAD23A EIF2S1 PEX11B HIPK2<br>ARL6IP4 ENAH NAP1L4 MSH6 OXA1L TMX1 TBL2 GTF3C2<br>HNMT TNPO2 RYK PRPS1 HSPB11 HDHD5 BECN1 VAT1<br>COPS3 MAP2K1 DSG2 RPA1 JAK1 HAUS2 CROT PHF1<br>GNA11 POLR2F ATP5MC3 UFM1 EIF2S3 TTC37 WASF2 SP3<br>CRTAP CDC42EP4 MPHOSPH8 KLHL9 HADHA COG5 NUMA1<br>GPN1 KLF9 FARP1 ACADM CNIH4 SH3GLB1 GULP1 TRAK2<br>ZSCAN18 CHMP2B FBXO7 SDHB C2CD2 CDC123 TTC17 WARS1<br>OCRL SDHA INSIG1 HBP1 RCN2 DENND4C TM2D1<br>SCFD1 CTNNAL1 PREPL NSMCE4A GNPDA1 ASMTL TMEM14A<br>MFAP1 ATG3 MRPS22 SNRPF TMEM106B FN3KRP NBN CFLAR<br>UBE2E3 RDX METTL7A ZNF217 CACYBP MPC1 RBFOX2 EIF2AK3

	LSM4 ATP6V0A1 OSBPL9 CDKN1A AP3B1 CCND3 KDM4B<br>MAN2B2 TRIOBP ZFAND1 SUN1 UBL5 DGCR2 NCBP2 ABI2<br>ISOC1 XPOT GNAQ USP1 CBX1 PPP6R3 PPDPF<br>CTNNB1 PI4KB OSBPL8 SLC25A37 TSPAN9 EIF2B1 AUP1<br>SCPEP1 SRSF10 IPO7 PCID2 NECTIN2 RBPJ USP22 HMGXB3 WTAP<br>RBBP4 PLPBP FBXW4 MANSC1 ACTN4 KRAS WFS1 SPTSSA<br>CEBPZ GPAA1 NUDCD3 DCTN6 TNFRSF1A NIF3L1 SGPL1<br>NUP133 ATP11B GMNN FGF13 MRPL9 APIP NAMPT UTP6<br>CUL4A C1GALT1C1 RALY PTBP3 MAP3K4 NFIC RPS6KA2 ALG13<br>RNF130 UBE2K RETREG3 PEX2 TLE5 RAPGEF6 EHF HPRT1 UPF3A<br>CLTB KHSRP TMF1 ABL1 ZNF532 CXXC1 LEMD3 MGAT1<br>STT3A PCNA PER2 ATG101 RNF4 BTBD1 TCTN3 RPRD1A<br>GLRX CTSL FOXJ2 MRPS27 POLE3 MFF ARL2 H1-10<br>TMEM168 RMDN3 PLOD1 AUTS2 PMPCA SLC35A2 TPD52L2<br>ABLIM1 ASAP3 RANBP1 OSBPL2 FASTK LAGE3 STAT2 ME2<br>YRDC SELENOT ARL2BP RBM3 NDRG2 P4HA1 PPME1 RHOG<br>PPIH TIMM9 MRPL22 EXD2 IP6K2 NEO1 ACTL6A CTSA<br>MRPS17 PCMTD2 LTBR C12orf29 IDH3G PEX10 DDX41 LMNA<br>TIMM10 HMGCR CLCN7 MCM3AP HDAC3 LGALS3BP ISCA1<br>TBK1 NCK1 SMAD5 MAP4 UBE2V2 CYBRD1 CCNH CCDC92<br>ITPK1 APPL2 UCHL3 CHCHD3 MTX2 HMGCS1 MAP2K2 LMF2<br>VPS54 MTMR9 TATDN2 ARFGAP2 NSFL1C LCMT1 HAGH KCNN2<br>CLDND1 SMYD2 GET1 RIPK2 RRBP1 CTR9 TNPO3 GTF2B<br>BSDC1 UNC45A PEF1 CHMP1B ATXN2 PTP4A1 KLHL24 SEMA3F<br>ABCD3 TMEM214 GMPS MAP7D1 MTRR DDX27 MPZL1<br>MNAT GPAT1 SMYD3 UBA2 AGPAT5 PRKCZ GPS1 ZNF350<br>UBR7 LONP1 TMEM33 FAM3C IER3 IFI6 IMPA1 PLD3 TOR1B<br>NDEL1 ENPP4 SLCO2A1 KIF1B ACTR1A TBL1XR1 TPRKB<br>SLC33A1 VPS4A VRK3 NELFB UTP18 USP11 NFX1<br>TOM1L1 NUP153 CBFB CD2AP STXBP3 SPRY1 CDC40 DUSP11<br>ZNF451 PAAF1 VPS72 MRPL39 SLBP BCCIP RNASEH1 SLC66A2<br>TRIM26 BCAP29 MED28 NMD3 UBE3C COLGALT1 USP48<br>TMEM165 ZNF672 PIGC BAIAP2 TFDP1 DNAJC1 FNDC3A RPA3<br>TAF2 NECAP1 MAT2B VPS51 STX6 SATB1 AP5M1 ZZZ3 MINDY1<br>EMC2 MAIP1 CHMP1A POP7 MED20 AKAP13 ARAF<br>RNF144A ANKRD46 SLC25A28 DOLPP1 MRPL13 TXLNGY GADD45B<br>CARS2 AVPI1 MAN1B1 OSBPL1A SKAP2 RNF10 TANK HPF1<br>NUP37 GRPEL1 NGDN CIAPIN1 WDR12 TOB2 TRIM37 KANSL2<br>AAMDC ABRAXAS2 PIGK ETNK1 RBM42 EIF2B2 SYT17 MX1<br>SEH1L RABGGTB EHD1 R3HDM2 CCDC59 TP53TG1 ILK PCNX4<br>SLC25A46 PDPK1 UBQLN4 SMAD4 CENPN GCA SKIV2L<br>CACNG4 MFN2 PRKAG1 POP4 TM9SF1 IDE PEX7 PPP1R10<br>PARP12 COMMD8 ZNF330 SLC3A2 CAND1 GAA BRD9<br>SDR39U1 MED16 MRPL34 HCFC1R1 ARPP19 CLOCK ALG6 ZNF277

	ITGB1BP1URGCP EXOC7 RAB23 ATG12 TXLNA SUPT5H BET1<br>DESI2 PRR13 MRPS28 RAB22A GSK3B LEPROTL1 NUDT21<br>RAP2A SCNN1A AATF GGA2 WAPL ADGRL2 ZNHIT3 NAPA<br>CFB SCAF8 NUDT11 CSTF3 EIF1AY GNG12 DIAPH2 PPP2R5C<br>MED23 B4GAT1 JADE2 SLIT2 STRN3 SEM1 UNG PDCD2<br>DNAJB6 TBCE TESK2 AFDN ACTR6 PPP1R14B PELO<br>NDUFS7 PIK3IP1 DGKD SF3B3 SUCLA2 SETBP1 AASDHPPT<br>GTF2F1 AKAP11 CLUH TRIM27 TUBGCP2 SMC3 DAZAP1 ZNF614<br>DR1 SLC35E3 ABCE1 C8orf33 FRAT2 ADAMTS1 RBM10 ALG3<br>GNG11 CLASP1 DIP2C RB1 MAN2B1 BCAM LRRC1 OARD1<br>RAD51C KIFBP CNNM3 PGD PPIE CKS1B HEATR1 PAF1TXN2<br>RCHY1 STAM RRN3 EEF1E1 WDR45 NTAN1 SEC23IP METTL5<br>NADK EFHD2 AP2A2 PIP4K2C PRELID3B P3H2 GBE1<br>ABHD11 TTC27 MBOAT7 TRIB3 DHX30 MTFR1 POMGNT1<br>PIK3CB SIVA1 MTREX LUZP1 NMT1 ING1 TRADD NXT1<br>TRAPPC11 MTMR14 SNX13 IFI35WASHC3 SYBU RNF24<br>MRPS30 FLII TXNRD2 MAP3K11SGSM3 TYK2 FAM234B MRPS18A<br>PSMG1 PPP2R5D APOO ANPEP ZW10 ZNF444 THAP11 SNX4<br>STX7CASZ1 ZNF384 FBXO3 MED27 R3HCC1 ASF1A KAT8<br>PSEN1 EIF4E MTERF3 PPP2R3C SORBS3 COA4 GCC1 NUP205<br>LIPT1 CRYZL1 MPP5 ITGB3BP MFSD11 CYBC1 SLC25A11<br>CYP27A1 RABEPK PSAT1 COX10 ZNF264 CELF2 NARS2<br>HSD17B8 PNO1 INPPL1 ZYX DESI1 PFKLZNF770 CLPTM1 CLPX<br>RANBP6 TRAPPC9 SOCS5 ACOT9 COMMD10 PBX2 LPAR2<br>TRAPPC2 CHPF ARFGAP1 NELL2 PIK3R4 ATP6V1C1 CYTH1<br>TGFBRAP1 IPO13 NFATC2IP B4GALT7 NBAS CLU HIP1R IRF1<br>DPY19L4 EPN1 CRAT RRAS2 PXN GART STX10 USF2<br>IL17RC ARRB1 ALG9 CRY1 INTS14 KLHL20 SQLE RNF146<br>SLC30A4 TIPRL DNAL4 TNIP1 FBXO42 ZBTB33 SLC5A3 RFNG<br>DDX49 SLC50A1 OXR1 ADSS2 ZNF562 SMARCD1 MRPS14<br>TAF9B RMI1 SLC7A8 ITGA8 C6orf120 SEPHS1 LARP6<br>GADD45G PPFIBP2 SLC35C2 AMTASCC2 EHD4 ETS2DCTN1<br>MAPKAPK2 BAP1 NDUFAF1 SLC25A14 GPR137 SLC25A17<br>DEPP1 TSPYL2 RFC5 EDEM1 TBC1D13 RC3H2 TNFRSF10B<br>RAB20 DCUN1D4 TBCD OAS2 MCTS1 RANGAP1 MBD3<br>ZBTB43 DLGAP4 ACAP2 MRPL11 SIRT1 NFYB COQ2 IFNAR1<br>ATG9A AGPAT2 CHIC2 ANGPT1 FASTKD3 KAT5 STX4IFT52<br>GEMIN6 TFAM SMAD3 MTF1 GMPPA KLHL12 CRY2 ROCK2<br>RAB11FIP5 SOD3 AFF4 DMWD ABHD3 NGLY1 SCYL2<br>MAD2L1BP CHMP7 NDUFA7 PDPR TRIB2 RPS6KA1 RPAIN<br>PARP6 ATF5PREP PRR5 TIMM10B PPP1R13B EEF1AKNMT<br>TM7SF3 CMAS TMEM115 WDYHV1 MADD NUAK1 ABCF2<br>CDK5RAP2 DMAC2L CSNK1G3 USP5 DGLUCY MCUR1 CCDC51<br>KMT5A SPHK2 BCOR LACTB2 PACS2 VEZT MGRN1 CXCR4

	STAM2 PTPN6 PXMP4 PPP1R9A VAMP2 RECQL CHD3 PLGRKT
	WDR73 CCS DCLRE1C RPS6KA4 ZBTB40 TPGS2 ILRUN ARHGAP12
	TMEM39A ACOX3 FAAP100 EXOSC5 SLC25A13 C5orf22 LRRC42
	FGFR3 DEF8 ZNF589 PRKD2 C11orf68 FOSB SDHAF1
	CCDC130 MSL3 IRF3 CXCL10 EPS8L1 ME3 CEPT1 PLCD1 MED7
	PQBP1 MFHAS1 BRCC3 ZNF573 ASAP1 UCP2 MRPL28 OCLN
	PPATSHC2 PELP1 TARP CRADD FARSA PKIA RIOK2
	PPP1R15A ECD PIAS4 RUVBL1 ROGDI YARS2 PSMC4 KLHL7
	SIGIRR NOL9 RMDN1 MOSPD2 STAU2 AMMECR1 UCHL5
	TRMO SPART ROBO1 PXDC1 OLFM1 CEBPA-DTMTF2
	FAM214B CHST10 PDE6D THEM6 PCTP HSPA14 OSTF1 SEC24D
	GTF3C3 MPV17 CENPB FASTKD5 C6orf47 LGR4 AMOTL2 TESK1
	TRIM32 MRPS12 MINDY2 TUBA4A SARS2 ZNF136 OGFR RPE
	SLC9A1 NLRX1 B4GALT1 SPATA2L SLC2A3 BDH2 PNPLA6 PPOX
	PCDH7 MED22 KRR1 UBFD1 GSTM2 ARMH3 CCDC91 SRBD1
	SSTR2 ITPKB DVL2 SUPV3L1 NDUFAF4 IFT122 AKAP5 DCAF11
	IFIH1 KLF5 CREBZF F5 FZD6 SP100 NIP7 CLMN IL4R
	SLC12A9 AP1G2 FOLH1 H4C8 SPAST GABPB1 CYLD VPS37B
	SMOX RBFA RMND5B INSIG2 DTYMK DLAT ASB1 NUP42
	ILKAP PVR RRP15 IL17RA GYS1 USP8 PPP1R13L SUPT6H
	GRIK5 NUDT2 ETV6 INTS1 ZNF580 POLRMT TTI2 FXR2
	COG4 EFS SKI MIIP PELI1 C2orf42 LIMK2 POLR3K SNRNP35
	ERCC1 NME7 DNMBP PLEKHM1 HLA-DRB4 UNKL RIC8B
	H2BC6 XYLT2 PIP5K1C BTBD7 YIPF4 FAIM SLC48A1 RET
	PEX13 MYNN MTO1 NUP160 IER5 ACOT7 BCL2 PPP2R3A
	SHFLGFOD1 PDE12 ATP9B SLF2 RTN2 GOSR2 MRPL2 SGF29
	EVI5 MCAT SLC27A3 BRIX1 TNS2 NOP14 SEMA6A THAP10
	PPP6R1 OGDH NCKIPSD PITPNC1 VPS26C SDHAF3 E4F1 SIRT5
	AMACR SPTLC2 DCK IPP SYTL2 PRMT3 PCBP4 RBMS3 TMUB2
	DHDDS MTG1 FAM102A SNTA1 EXOC3 KLHDC4 KAZN
	NADSYN1 C19orf54 HIF1AN IFNAR2 TMC6 ANKZF1 PRKAB1 PKP1
	PLXDC2 RPAP1 MYO9B ARAP1 TLR5 GRWD1 COL8A2 TRABD
	CYHR1 NMT2 TSSC4 KCTD9 ITGA2 BCL11A MEN1 SPN
	NUP214 TMEM209 DHX9 PCYT2 UEVLD MORC3 RPAP3
	MBIP TAF6 EML3 BCORL1 PCSK7 TRIM5 KAT2B ELMO3
	GNL3L GIT1 PLEKHF1 TBC1D1 PEX1 C1orf216 TMPO NR4A1
	LLGL1 PTGER4 ITPKC FBXO31 BRPF1 KIAA0513 OTULINL TAF1C
	OSTM1 ZNF654 HECTD3 PIGQ MRS2 CLASP2
yellow	RPS15 ACPP KLK3 RDH11 NKX3-1 TACSTD2 P4HB SORD
	KRT18 MSMB TMBIM6 SARAF TMEM59 ATP6V1G1 NPY CDH1
	SSR4 ABCC4 CNDP2 GOLM1 TMEM87A AZGP1 SPOCK1
	SERPINB6 TMPRSS2 AMD1 APLP2 CPE SC5D HSPA5 DDAH1
	TSPAN1 CIB1 TMED3 TM9SF3 BNIP3 H2AJ GLUD1 C1orf116
	FOXA1 ATP1A1 DHCR24 PMEPA1 CANT1 CALR KIAA1324 ACLY

	ARID5B FUCA1 DSP NDRG3 SMCO4 DCXR ARCN1 OR51E2<br>ACSL3 NEDD4L TMED9 STK39 ST6GAL1 SOCS2 XPO1<br>ARFGAP3 UAP1 SH3BP4 MAP7 FASN ERBB3 NAAA<br>SLC44A4 SND1 SLC7A1 TRPM4 LRIG1 ARG2 C1orf115 UBE2J1<br>CYB561 MYBPC1 SLC35A1 KRT8 RAP1GAP ATP2C1 SEC22B SDF4<br>TMEM30B CCNC SCP2 CHP1 CORO1B CLDN3 AFTPH<br>QDPR FAM174B NCAPD3 TRPM8 MARC1 IQGAP2 HOMER2 PPM1H<br>HYOU1 ANXA3 ENTPD5 PRSS8 GOLGA5 GMDS PGM3<br>SLC35A3 SUOX ALG8 PAK1IP1 MBOAT2 TRIM68 GMPR CNPPD1<br>HPN FBP1 ERLIN2 CCDC47 ST14 ASTN2 DSC2 PRKCH CRYL1<br>ADRB2 RWDD2A DPAGT1 ABHD2 EPHX2 GCAT GNE CDS1<br>PITPNA SLC2A10 REPS2 PTPRN2 SLC35F2 PDZRN3 TCTA TMEM254<br>PNKP STEAP4 BCAT2 SLC1A5 IVD TTC38 TM9SF4 DHTKD1<br>OCEL1 RAE1 GREB1 CUX2 TPMT GEMIN4 GJB1<br>TMEM268 WIPI1 AP1M2 OVOL2 ABCC5 PART1 AACS<br>TFPT RAB3B CPT2 KBTBD11 BCAS1 MAGEF1 GSTZ1 ORC3<br>CTNNBIP1 KIAA0319L GNMT PGAP6 GPRC5C CYTH2 MOGS<br>DOP1B CNPY3 IL20RA POLD4 PAFAH2 KLF15 CDK2AP2<br>BCKDHB ESRRG MACO1 RRNAD1 MMD
blue	RPS23 OAZ1 RPS4X RPS9 DSTN CCNI COX4I1 PARK7 RPS4Y1<br>HUWE1 ARL6IP5 RPS7 CD63 CIRBP RHOA MGP TSPAN8<br>DBI GDI2 PFDN5 SEPTIN9 CUTA RPL37 NPC2 RPS27L<br>IMPDH2 ECHS1 HEBP2 ST13 TNFSF10 GPX4 CLIC1 EIF3D<br>SRSF5 CCNG1 TM9SF2 SMS SPINT2 NGRN TUG1 STK24<br>LAMP2 ELOB ALDOA SCAMP1 CLTA FLNB LITAF DDOST<br>RPS11 TCEAL9 DDX17 HMGB1 H1-0 BCLAF1 MAT2A SLC4A4<br>AKR1A1 CMPK1 SH3BGRL ATP6V0E2 NDUFA13 RPL27A CYP1B1<br>PSMB3 YPEL5 NDUFB11 FDFT1 ACSL1 ATP5IF1 ASAH1 AP2S1<br>ADAR PNN COMT TSPO PGM1 RBM47 CD164 CEBPD<br>ATP6V0B TOB1 PCMT1 PSMB6 EIF3G RBM5 PPP2CA<br>WDR83OS OGT AHCY CETN2 RNF114 FKBP11 AURKAIP1<br>NDUFA8 SF3B5 BRD2 TUFM MKRN1 STUB1 SUCLG2 RTCB<br>GPX1 COPZ1 PTPN11 MYDGF SERINC1 ZMIZ1 RBBP7 CSNK2B<br>FAM162A VKORC1 MFSD1 SON RERE SGSM2 PTPRO ARGLU1<br>UBA1 DDX3X CENPX TXNL1 HEBP1 CCT7 AKR7A2 ACAA1<br>UQCRC1 SH3YL1 MRFAP1L1 YTHDC1 NRIP1 ENDOD1 SRPRB<br>DHRS3 DNAJB9 TPD52L1 FIS1 CTBP2 NDUFB4 CHMP2A METTL9<br>MAGED2 OGA ADH5 PRRC2C SAR1A GORASP2 FXYD3 SDHC<br>ALG5 SNRNP70 EIF5B SMIM7 CDC42 SRRM2 LARP1 BEX4<br>HADH CAMK2N1 PUF60 PPP6C PLIN3 DCTN2 DGUOK<br>HNRNPUL1 GLUL CIAO2B CERS2 DHX32 NCOR2 AP3D1<br>ARPC1A RO60 RAB25 VAPA TBCB CAPN2 CTBP1 TSPAN6<br>SURF1 NFIX MRPL17 IGBP1 ATP6V0D1 PERP HNRNPH3

		SEL1L3 MAPKAPK3 TPP1 CSDE1 PPCS DPP4 IMP3 RBX1 OLA1 PSMD1 TRMT5 SARS1 SNX17 YIF1A RBM39 CHD4 MYLIP EIF4G1 YIPF1 MAP3K5 MRPS15 NDFIP1 HSD17B6 DCTD RSL1D1 SLC25A36 USP34 COPS6 TPR PHB GRHPR NKTR POLR2I WSB2 MIOS FNBP1 MRPL40 PPP1CA MRPL57 APRT GOLGA3 SAMM50 IFT20 LUC7L3 PCM1 AR KIDINS220 EIF4E2 ZNHIT1 TMEM134 TST POLR2L WSB1 DDX24 NCOA1 SCAND1 NUP43 RASSF7 KCMF1 HMGN3 KMT5B CCDC28A N4BP2L2 SMPDL3A TCEA1 YES1 TBC1D9 TFRC GLOD4 CRBN PHF3 NOTCH2 MYH9 NOTCH2NLA DERL2 SDC4 WLS MACF1 SLTM UBR4 IDI1 EIF1AX HOXA10 RBM25 IFITM3 PPP4R1 BCL6 KDELR3 HSPH1 SRRM1 LETMD1 RAMP1 AK4 TINF2 PIEZO1 PRPF40A ERGIC2 MB MBTPS1 COPE PGLS YY1AP1 TMX4 TMEM208 C2CD5 MSMO1 UBE2Q1 INPP5A KHDC4 THBS4 COL4A5 KDM3B CLK1 CTSO CD2BP2 RALGDS AFF1 ILF3 SNAP23 PPP2R5E RXRA KLC1 VGLL4 RIPOR1 SDAD1 SNRPE APEH SNRNP25 SQOR UBP1 PRR15L TBC1D8 MBP RBCK1 MAGEH1 TAF15 BRD8 SF3B4 PDHA1 EMG1 PCYOX1 TMEM50A CLSTN1 PINK1 GDI1 ELAVL1 EMC6 ROCK1 C12orf10 TBC1D9B DDIT4 ZNF266 PAK2 USP47 E2F4 XPO6 PLOD3 MINDY3 SCCPDH IER3IP1 PURA BANP USP33 EIF5A CDC37 PSMD13 RNPEP TOP1 RPF1 PIGT MAX KYAT3 PIK3R2 MKLN1 CARS1 USP4 OPHN1 NCOA6 GNL2 PITRM1 ELF2 HAT1 POLR2C GLRX2 TXNDC9 TDG SNX2 SCMH1 EIF5 MTHFD2 PKP4 ADO SUGP2 PCCA CNTNAP2 PEPD DROSHA PRR14 CRK ANXA4 RNF14 CHTOP CRYM DDX50 RNF13 MXI1 ACAT2 SERTAD2 NEMF HGS SECISBP2 PRPF31 PFDN1 GOLPH3L RETSAT ARHGAP6 TUSC3 TAOK1 SELENBP1 CELF1 SETD2 COQ10B CRYZ RAB27A GGA1 PRPS2 BTAF1 GMPR2 SNRPC MED13 BABAM2 ACOX1 CDK13 NUP88 FAN1 TIMM17A MRPL41 PDXK UPF2 CBX6 AAK1 ATP8B1 PRUNE2 DDX39A BMS1 CEP350 FBXO11 BMPR1A TMEM135 FRYL BBS1 ZNF160 SPEN CUL3 CKAP5 RNASE4 ZMIZ2 SAFB2 ZNF419 PHACTR4 WNK1 ARHGEF18 IFRD1 PPFIA1 TCTN1 RELA SCAP KBTBD2 LRRC41 STX16 GCNT1 CHKA CAMSAP2 MRPS11 GIGYF2 YIPF3 LAMA5 DNAAF5 PRR11 TENT4A BAZ2B UPF1 UBR5 NOP2 CDK7 SLC25A4 TBPL1 MARF1 TOMM34 ACTR2 PMM1 MCM7 ZNF32 ZNF146 THYN1 RUFY1 FAM13B CHERP GCC2 KIAA0232 NR3C1 PPP1R2 FAM13A SMG7 ABR PUM3 BPTF MBNL1 MPST GIPC1 DHPS MTUS1 TMED1 POLR2A DLG1 HSP90B1 TIA1 N4BP1 TNFAIP1 HSDL2 PEMT COPS8 UBE2I DMTF1 ATRX IPO9 UBE2A QKI PRKRA ZDHHC17 TEX264 CD151 PXMP2 ZNF544 EXTL2 RXRB RBM6 SCAMP2 KIF3B GAPVD1 PSMD5 ARID4B

HERC1 CLN5 DAPK1 JARID2 ZMYND8 TES UBE2B PBRM1
KLHDC10 GOLT1B AGA NSUN5 NEK7 SMARCC2 TTC1 SCAF11
PPP1R12A UBAP2 GRINA TP53BP1 AKAP12 CYB561D2 RBM26
DOLK PRDM4 POGZ SRPK1 KDM5D UBE2G2 CLK2 PTPN1
TOMM22 KAT2A DST PEX19 GCN1 MTSS1 IMPAD1 DENND5A
SPIN1 GATAD2A GOLGA2 MAP1B LAD1 GMEB2 TSN TSC1
SETMAR ADCK2 MYO1B TMEM50B NFAT5 ANAPC15 SLC20A1
DHRS7B MTMR4 AKT3 NCOA3 MGMT SIRT2 ZNF432
SLC35A5 IVNS1ABP PRKCA THOC2 SCUBE2 F2RL1 SOBP
CLK4 UNC119B GRB10 DDX3Y DNAJC10 KIAA0556 UFSP2 PKM KHNYN
FBXW12 THOC1 CHORDC1 MZF1 CITED2 ZNF337 PHF2
PSIP1 ZC3H15 MTPAP MIS12 METTL17 SLC35D2 TRRAP
PAFAH1B3 STK38L SOAT1 APH1B RAB14 CRNKL1 SLC12A7
TCERG1 MLX HPS6 HCFC1 TTF1 MTMR6 INTS3 CDK17
GUCY1B1 DHX16 AGRN SUPT4H1 WDR59 DIAPH1 RBBP6 USP16
CLPPDIP2A SNN LAMTOR2 ATP9A DUSP14 DKFZP586I1420
AZI2 ZMYM3 PIGN GORASP1 DFFA ARHGAP17 GRB2 MARC2
CALCOCO1 EIF4G3 PNRC1 HDAC6 BICRAL AP3M2 WASHC4
SP110 ITSN2 CPSF7 ANKRD10 LANCL2 UBE2NL MVK OGFOD1
EXOSC10 MRPL48 BIK CAB39L ZNF587 LMAN2 EXOSC4 ZNF611
ZBTB5 STIP1 SMC1A PARP2 MGAT5 HMBOX1 AIMP1 MTR
R3HDM1 OTUD4 ERAL1 LIFR NOL8 AHI1 PTBP2 AKAP7 CPQ
ZFAND3 ADARB1 CHFR CAAP1 INTS6 ZBTB18 DEDD UPF3B
MKNK1 ABCC1 HARS2 ZRSR2 TNRC6B RAB11FIP3 SEMA4A
TUBG1 RWDD2B PTCH1 DNAJC7 DNAJB14 IKBKG CDC42BPA
RAD17 ARIH2 ASXL1 DNMT1 ANKRD27 PHC2 SMUG1
ARMCX5 ADCY6 CFAP69 PHF21A RNF220 NECTIN3 KIAA1109 CASP9
CNOT4 ATG14 NEK9 WWC3 ATM IKBKB SIRT3 SAFB
RFK IFT46 MAZ WIPF2 KMT2A IFT88 STK4 RPRD2 CCNL1
MAU2 ASB8 SLC39A8 PAX8 PMS1 PTPN12 WDR19 TARDBP
SETX FKBP9 EPS8 FBXL12 MAP4K5 TNFAIP3 SSH3 CBLB
DNAJC13 TCF7 PFDN2 ZNF692 ZMAT3 LPIN2 SETD5 JAG2
THBS1 PTPN18 ZNF12 SLC5A6 PIP4K2B MINK1 TLK2 PIGH
CUX1 ABHD4 GTPBP3 CPT1A HELZ COX11 AKAP8 NAB1
APPBP2 VPS11 PMS2P1 NPC1 VAMP4 LARP4 ASAP2
TBC1D10B RNF44 PUS7 PRPF3 CHD1 ATG13 DDIT3
TRA2A RABEP2 BTD TRMT61B MID2 SEPTIN8 CIAO1 MTA1
ANKRD12 SETDB1 VPS13B YEATS2 ICE1 GLE1 ETHE1 MLXIP
MED13L CAPRIN2 BNIP2 TOPBP1 NIPAL2 ZNF362 ZSCAN32
SLC27A2 SUCO BICD1 ZBTB11 PIAS2 PAXBP1 XKR8 FBXO46
DCAF16 ACVR2A LYRM4 ASH1L PDE4D NCOA2 KLF10 H2AX
WDTC1 EP400 AGFG1 PDK3 TMEM184B RFC1 C7orf26
ANP32E DNAJC24 STAT5B STAG1 SUMO4 SFSWAP SCML2 SF3A2
ZC3HAV1 GPATCH8 BRD4 C1orf50 TOR3A LARP7 ANKRD28 UGGT1

	TSC22D2 HGH1 RECK TULP3 CD83 RAD1 ATG2A HNRNPF TRIM38 SH3BP5 ULK1 ATRN EAF2 RUFY3 MRPL52 ZZEF1 FASTKD1 PRRC2A TCF3SDC1 BAD RTN3 EDEM3 SYNE2 DBN1 FOLR1 SCRIB ERBB2 SNX6 UMPS HIPK3 RPP38 TUT4 RAP2B TBCC FADD USP32P2 FAM193BSART3 MRPL19 ASPH RAD50 BRD1 PCGF3 FUT6 ZNF26 MUTYH FGFR1 AMPD3 UBE3B ALMS1 ITGA6 MAPK3 LUC7L ANKRD11ZC3H13 NAA16 SPOCK2 EZH1 KIAA0930SNAPC4 GTF2IRD1ZBTB1 PID1 SMAD1 MINPP1 PODXL YIPF5 FAM117ATPCN1 RBBP8 ZNF93 SLC35C1 TNK2 RABL6 PIGO DDX6 TAF4IRF7 BAZ1B C14orf132 ISG20L2 THUMPD2 PRDM2 CEP170B ZNF226 NF1 USP32 ZFC3H1 RESF1 MAP3K3 KAT7 ATR SLC25A12 UNC5B MDN1 RMND5A TASOR IQCB1 SIAH2 DIDO1 SCO2 NME5 FGGY SUPT20H SENP5 CD40 ARHGAP35 NIPBL CENPBD1P1 PTPN2 OAZ3 SH2B2 RCBTB2 ZBTB24 TACO1 PAFAH1B2 CHD7 PLCG1 SBF1PLA2G6 MVB12B STX17 LSG1 ARHGEF16 ATF7IP TCF20 RBM14 IGHMBP2RCAN2 FBXL14 WNT6 SOGA1 ATG4B KDM5A FAM53B TBC1D2B PRKACA ZNF85 SULT1A1 RGS3 WDR3 RLF NSD3 POLR1E MUS81 PTCD3 SAP30 UGGT2 ERI2 DDX11 ELK3DRAM1 SRR ODR4 LRCH3 EDIL3 KLF3CCP110 ING3 MITF TRIM21 GSK3A RABGAP1L FBXW11 MZT2B GON4L ST3GAL5 FBXL7 AKAP10 ZBTB7A CCNT1 SMC5 MOSPD1 KDM4A POFUT2 WRAP53 USP21 PHLDA1 MET STRADA VASP SCD5 SSBP3 MTAP INSRABCA5 PIP5K1A ZCCHC14 REXO4 CTPS1 ZNF443 CUL7 RPP30 MAPT POT1 TTI1 AGGF1 C11orf95 PCLAF EDEM2 VPS13A PHF20L1 ZFYVE26 PTPN9 CYP2J2 PLEKHM2BTN2A1 DZIP3 KIF13A MMP14 PLXND1 ANGEL1 CROCCP2 SMC4 NDE1 RABIF CDH10 RAB6B ACOT8 PPP1R3D WWP2 SSX2IP FHOD1 SIX5 MARK3 PHC3 RUBCN PLPPR2 FMO4 ZNF212 ZNF318 MRTO4 BPHL PRKAB2 NOTCH1 PARD3 SSH1 EXPH5 NUP210 LRP6EED ZNF529 ANKRD36B AHCTF1 FRMD4A HBS1L PWWP3ATPP2 ESF1PFKFB3 CRCP JRK TNKS2 KLF7MLLT10 TAF1MPI E2F3SP2 XAF1 PPP1R26 MAGI1 CDC27 PPP4R3A RBM28 PI4K2A CSAD MAVS THSD4 ZNF248 AATK ACIN1 ABCC10 LMBR1L GOLIM4 TTC31 SKIL GAS8 PHC1 LNPEP TMEM131L PLCXD1 DOCK4 HEATR6 MAFG PRKAR2B TMEM260 ZNF507 CWC25 PLAGL2 GPRASP1 PEX1
green	HLA-B COL1A2 COL3A1 VIM HLA-DPA1 IFITM2 HLA-E SPARC LUMCD74 HLA-DRA LGALS1 CTSB COL4A1 ANXA2 COL6A3 HLA-F SRGN NREP LASP1 FN1 ARHGDIB IGFBP3 COL1A1 IFITM1 LAPTM4BCRYBG1 GJA1 GPNMB EMP1 ABHD10 HTRA1 S100A6 TGFBI CTSKMXRA5 COL6A1 HLA-DPB1 DPYSL2 PAFAH1B1 RAB31 PLAAT4 KCTD12 TNS3 PPIC LAPTM5

	HLA-DMACOL4A2 SULF1 APOE PDGFC VCAN PMP22 YWHAH<br>S100A4 POSTN TGFBR2 LTBP1 MFGE8 AKR1B1 SEPTIN11 ABCA1<br>S100A13 OLFML3 DAB2 PLS3 CLIC4 MAFB ENPP2 C1orf54<br>APOC1 CDH11 COL15A1 ARPC1B MS4A6A RFTN1 LHFPL6<br>ARHGEF2 TRIM22 TAGLN2 CD93 COL5A1 THBS2 CORO1C TYROBP<br>PECAM1 RAI14 SLC39A14 PCOLCE FCGRT C1QB TNC IFI16<br>HEG1 CD53 RAC2 SERPINE2 FBN1 SPP1 WAS COL5A2 CSF1R<br>PDGFRA PLIN2 RSU1 MRC2 ITGB2 MXRA8 FRZB<br>SERPINH1 CHST15 SEC23A NNMT CHN1 RNASE1 FXYD5<br>ANXA6 AQP1 GLIPR1 SNX7 COL8A1 CTSC LEF1 MMP2<br>GLT8D2 GSDME FYN P4HA2 PLBD1 THY1 HCLS1 ZEB1<br>HEY1 SPATA2 CYBA GIMAP4 AP1S2 FGL2 SLC9A6 CCL5<br>LAMA4 PLSCR4 ECM2 NOTCH3 LMO2 AIF1 TPST1 ADA2<br>CD14 ALOX5AP AOPEP ENG TFPI MMP9 COMP DPYD DSE<br>EMP3 PLP2 DYNC1I1 JAM2 C1QA SLCO2B1 OLFML2A IL2RB<br>ENTPD1
black	EEF1A1 ACTA2 TAGLN ACTG2 MYH11 TPM1 MYLK DUSP1<br>TPM2 NIBAN1 HNRNPAB HSPA9 SYNM CD44 NEFH<br>ADIRF ZFP36 STAT3 PCP4 MATN2 SHMT2 SORBS2<br>TIMM8B TMEM106C TIPARP NDN PDIA3 HOXC6 ITM2C APP<br>KANK1 HDGF MYL9 STAT6 PAGE4 FHL2 MPZL2 HMGB2<br>RPLP2 MCCC1 PYGB NR2F2 LPAR6 MEIS2 FOS MT1H<br>STX12 COCH PLPP3 TMEM97 LTF KLF6 ZNF331 VPS52 CHD1L<br>CALM1 SSPN SPOP PHACTR2 DNAJC9 HSPB8 PBX1 VWA1<br>NEDD9 MAOB CCDC25 SCRN1 ASNS EGFR MCM6 MBNL2<br>PPP1R12B DKK3 ZNF185 TRIM2 WWTR1 KPNA2 PAMR1<br>CDC42EP3 CEACAM1 ISL1 PKIG CKS2 DIXDC1 PIGF CHRDL1<br>WNT5B LMOD1 GAS1 OSR2 NUP85 PDE4B KRT15 KCND3<br>RBPMS TRAF3IP2 HILPDA RBM4B CDK2 SPINT1 CDKN1C CBX4<br>IRS1 FEN1 PTTG1 ADGRG1 MEST ATF3 PLN TCEAL2 OBSL1<br>C1RL RAI2 FOXF1 MCM2 RFC4 SLC39A9 LPCAT1 HMGB3<br>UBE2C VPS35L ABCA8 KRT5 LDB3 ZNF430 OLFM4 STMN1<br>PEX14 TCF21 TRIM29 SEC22A FERMT1
grey	RPS10 RPS28 RPL7 RPS18 RPS27A RPS24 RPL6 RPL11 EEF2<br>UBA52 TMSB4X PABPC1 GAPDH PSAP EIF3E GNAS CFL1<br>RTN4 CD81 TXNIP PEBP1 MTCH1 ANP32B SEPTIN2<br>TSC22D1 MLPH EDF1 ATP5ME YWHAB ENO1 PRDX6 BASP1<br>MT2A TUBB TRIB1 FTH1 SERPINA3 YBX3 HLA-C RPN2<br>REEP5 ALCAM MAOA NARS1 ARPC2 CTNNA1 CAP1 GNB1<br>DBT TUBB4B TM4SF1 RNASET2 SRSF6 CST3 LDHB PLA2G2A SERBP1<br>NDUFB8 MLEC PABPC3 KPNB1 PTPRF KARS1 MT1X<br>SQSTM1 PDLIM5 MT1G GNS ZFAND5 CKAP4 TRA2B RCN1<br>CKB GOT2 CAPNS1 SRSF1 ACTR3 SCD APOD HNRNPM RAC1

	ATP1B3 LEPROT SEC31A CDK19 UBXN4 TUBA1A STAT1 CD47 S100A10 GATD3A FBL TGOLN2 IFNGR1 H2BC21 CS HSBP1 SLC35E1 TOMM70 NCL PRCP PTMA LCP1 CBX3 VAMP3 NME4 MT1F BUB3 IFNGR2 GUSB ACACA IST1 PRPF8 DENR VPS37C NONO COBLL1 PSMD4 EGR1 CAMKK2 SLC38A2 LRP10 ZNF91 ITGB5 PJA2 USP9X STAU1 GARS1 ALDH3A2 MSRB2 CREB3L2 PSMC5 SORL1 LGALS3 MAPK1IP1L EIF3A SEC62 GPD1L KIF5B AHCYL1 CRELD2 H1-2 GOLGB1 SAP18 SEC61A1 DAG1 VPS28 CAPRIN1 WWP1 ECH1 ATP6V1E1 PICALM AHSA1 ALDH6A1 JAG1 GALNT11 TOP2B CHSY1 FAT1 FMOD PAICS RHOBTB3 DNAJC8 METAP2 SUZ12 PTK2 ISCU IDH2 CAPZA2 SPG11 GUK1 SINHCAF PSMD2 FKBP4 NELFCD CTSH HLA-G SERINC5 FAF2 NUCB2 HNRNPDL UBE2L6 H2BC5 SPATS2L RRM1 MAEA IDH3B TRIM44 SRSF11 VCP EID1 ATP5MC1 OSBP EBAG9 C19orf53 SMARCE1 CNOT1 SYF2 LYRM1 FAM111A MCCC2 HDDC2 PPIB TERF2IP PABPC4 DMXL1 UBE3A CDC16 BBX UROD NDUFV1 RYBP PPM1B PRSS23 SLC11A2 EIF2B4 RAB5B NT5C2 PAPOLA WBP2 PARL ALDH7A1 CNOT2 NDUFA10 CUEDC2 SRPRA HLA-DMB SNHG32 BUD31 CCDC6 SEPTIN7 IL13RA1 PSMD8 MMP7 ALDH2 BACE2 PHIP U2AF1 KTN1 MEA1 CERS6 FEZ2 SNRPD3 APH1A XPNPEP1 GRN ATP1B1 HES1 BTG2 LRPAP1 ATIC NAXD SFPQ PHGDH BCL2L2 ANK3 EIF6 S100A11 RGS5 LRRC47 TBX3 AQP3 SACM1L CD59 NCSTN GSTA4 FAM171A1 RAB8A TSPAN31 TPM3 ARID1A TTLL12 AP2M1 FXR1 EPM2AIP1 UBE4A NDUFB1 GSTK1 TRIP12 NME3 GANAB WBP11 ID2 TLE1 C11orf58 ATP6V1B2 PPP2R2A FZD4 RBM22 SEC63 DNAJB1 GALNT7 COPS7A TKT PHYH RETREG2 CADM1 IK EHBP1 TMEM43 BLVRA SECISBP2L RAP1B IQGAP1 EFCAB14 COQ9 SESN1 ANGEL2 COBL CCT6A IL6ST OAT PTEN TMEM30A ATP5F1D DAAM1 EIF2S2 HNRNPA3 FBXL5 MLF2 ZNF706 CTDSP2 ZBED5 IRF6 CAPZA1 THUMPD1 KRCC1 EFNB2 CSRP2 POLD2 RPA2 RIN2 VPS26A FOLH1B ADPGK RNPS1 ENOSF1 FBXO9 KPNA4 BCL2L1 RASA1 ZBED1 SHLD2 TMEM131 ACO1 SLC38A1 DCAF7 FAM98A TSTA3 TRAPPC3 CYB5R1 KLK11 HOXB13 VPS41 LDOC1 FKBP3 SIL1 ATXN10 RALA TEX2 KLHDC3 NFKB1 RHOT1 COQ8A TFG NAGK FAM168B CSNK1D GID8 ENC1 TIAL1 COPS2 ESYT1 DICER1 ZDHHC6 ADAM9 PTPA UBE2D4 IGF1R CADPS2 TNFRSF21 ITGAE BHLHE40 PSME4 CERT1 CFAP298 RHOQ ARF5 SEMA3C IGFBP2 SLC9A3R1 CALCOCO2 CFDP1 DNAJA2 SPATA20 BAG6 PRMT1 AKAP1 TWF1 LTN1 SDF2L1 SNX5 TXNDC15 RB1CC1 GTPBP6 TRIM28 GOSR1 FAM89B PDS5A EPS15 TCF12 ACO2 NSMAF WWC1 GHR U2AF2 JMJD1C PRKD1 GSS MTX1 NQO1 MTIF2 PEX5 ADD3 UBQLN2 GNB2

	ARHGEF3 FDPS UGP2 LRBA TXNRD1 SDC2 UBE2D2 GTPBP4
	PDE9A ZNF106 KIF13B IBTK TRAP1 STK3 MSRB1 TECR TDP2
	FIBP ITPA LAS1L KRT19 JUP CRKL PRKCI IRF9 FKBP2
	UNC13B WBP1L DUS1L KDM5B PDLIM3 PEX11A TUBB3 EXT2
	RRAGD ATOX1 DNAJA4 ZNF395 TMEM70 EPB41L4B SLC25A38
	MIPEP PIAS1 VTI1B ASXL2 NOSIP FAM114A1 FAF1 UBE4B
	DNASE2 LMAN2L GALC TMC5 WDR26 TAP1 PRKAR2A TEX261
	URM1 PAK4 WASL SF3A1 TRAPPC6A ZNF274 QRICH1
	FBXO38 TCAF1 SLK MEAF6 RAB1B PANK2 TRAPPC2L PGAP2
	SPR KLF4 GNPTAB SGK1 BBS4 GOT1 VEGFA NR2F6 PIN4
	KCTD20 LXN MRTFB PEX16 TSR2 DUSP22 B4GALT5 MAPK10 CREBL2
	CYFIP2 PRMT2 ATP8A1 NMRK1 DNM1L LYPLA2 COPG1 MAF
	ANG UGCG TRAK1 RAB11FIP1 RALBP1 FAM172A ARMCX2 LBR
	TBC1D4 LSS MYL6B MTCH2 CCNB1IP1 ELF1 ARFGEF1 CTCF
	SLC26A2 RALGAPA1 PRKCD SREK1 LRRFIP2 ABHD17A UBR2
	BANF1 RPS6KC1 DDX46 YAP1 SLC37A4 TBC1D16 IDS GOLGA4
	PROS1 BCKDK SNRPA CCDC86 PIN1 INTS8 DCTN5 ATXN1
	CHCHD7 PRPF4 RPS6KB1 TXNL4A TERF1 USP10 SNRK ADRM1
	RGCC STAP2 SZRD1 PNMA1 HERC6 ASS1 DENND1B
	MKKS NUP50 MRPS10 DCP2 SNTB2 ENTPD6 RNF167 SORT1
	WASHC2C HOOK1 PDSS2 ITPR1 ACVR1 ZBTB16 ARHGAP32
	PSME3 SLC30A5 SMARCD2 RCOR3 TJP2 F3 INTS12 PPP2R5A
	TSPYL4 C16orf58 RNH1 SLC2A1 BLVRB PRKDC AAMP RFX5
	PLOD2 ATMIN TSPYL5 IFRD2 TLCD3A TADA3 MIEF1 GCLC
	TYW1 UROS CRELD1 MTMR2 ZMYM4 RNF187 MID1 SIM2
	PSMB10 ZNF24 BCKDHA DIO2 ILVBL DHX29 SNW1
	FAM189B TMEM222 RNF34 GTF2E2 CCT5 MAPKAPK5-AS1
	ANKRA2 ICMT REV1 GSTT1 ARPC4 INHBB GDE1 AQR
	BTG3 RTN1 SEC14L1 MAPK1 TUFT1 ARMC1 STX3 MAP1LC3B
	EDRF1 DDHD2 NFIL3 PIGR ZHX2 MAPRE2 EEF1AKMT3 PMVK
	ORMDL2 SMAP1 CSK SMNDC1 RBM12 ELK1 PLA2G12A EML4
	ALDH1A1 ANKRD49 STX5 SREBF1 RAB5C POGK UBE2H CDC23
	KCNMA1 BAG5 PLEKHA1 JADE1 ELF3 SLC41A3 TNFRSF14 NCALD
	KXD1 DCTN4 TCIM FLOT1 MED1 PLAT GAR1 USP13
	ANKRD17 PKN2 MRPS18B PSMD11 ZC2HC1A CYB5B VOPP1 ERLIN1
	SNX27 HLA-J PAQR4 FECH SLC22A5 MAN1C1 SRM RSBN1
	ZCCHC8 CEBPA ARSD TSR1 LYN SLC38A10 PGC RBM15
	SOWAHC BMPR2 ALOX15B EPHX1 ORAI3 AIDA DDX10 CBR3
	RPS6KA3 NQO2 DNPEP BCS1L AGL TFAP2A DEXI KPNA3 TRIT1
	SLC31A1 GSPT2 CBS CREB3 ETS1 RNMT ACSL5 STK19
	MRPS16 PTPRM IGSF3 ARFGEF2 NRP1 C9orf78 SMARCA5
	WDR11 FEM1C MTHFD1 RPIA PUS3 SNAPC5 SRPK2 TRIM36
	HTATIP2 CPA3 DPF2 LRRC8D IGF1 AP2B1 VPS45 HS1BP3
	SHROOM2 SPAG16 GTPBP8 NEU1 UFD1 NAA10 ZNF593

	MRPL12 WDR48 EPHA3 PYROXD1 PTP4A3 EMDERMP1 FANCL<br>PPP1R3C THOC5 ID3 STK26 AGK MFSD5 PSD3 MICAL2 TULP4<br>CPNE1 BAMBI WDR77 TMEM223 FABP5 PDS5B DACH1<br>ARHGAP29 ARL3 CCN3 TFCP2 PRUNE1 MALT1 SNX11<br>HSPA4 TERF2 POLR3B MAN1A2 RIPK4 OPA1 ARMCX1 MME<br>LMAN1 TRPV6 NECAB3 AP1B1 BAZ1A ESRP1 RNF138 LIN7C<br>UBL4A PLCB1 COIL SLC30A1 DEPTOR SALL2 PRPF6 MRPS31<br>GTF2H3 ZC3H7A VWA8 HMBS ZC3H14 NUP62 CUL4B DYRK4<br>SGMS1 TMEM243 CLN3 CEMIP2 ABCG1 PIK3C3 KPNA1<br>ATF6TSKU DDAH2 OGFOD3 RABEP1 CDC5L HLA-F-AS1 SLC1A4<br>DECR2 CDS2 SS18L1 TEX10 CSNK2A2 FZD1 DUSP12 SNX19<br>ABITRAM DNAJC3 PSEN2 PPM1A ENTR1 DDX23 MED4 FBXW2<br>GTF2H1 SMPD1 ZFYVE16 MED8 PLA1A COL9A2 HECA SAP130<br>RRS1 CCNT2 RLN1 UQCC1 SPAG9 ARIH1 GFM1 CXCL14<br>TFAP2C MAP3K2 ATG5 MON1B RGS1 CEP57 ESR1POLDIP3<br>CLTCSNRPD1 MPPE1 NUTF2 C9orf16 ELL2 C18orf25 TRAM2<br>ZNF271P LSM12 EFL1 UBE2M ARHGEF5 PI15 HS2ST1 PCF11<br>RAB11FIP2 IDH3A EAPP ADGRF5 MACROH2A2 MCRS1<br>TSPAN14 LPXN DPP3 CSGALNACT2 SLC25A20 IMPDH1 NIT1<br>SLC17A5 RORC GSR PIGVUBA5 MAPK13 FARS2 POLR2K SNAP29<br>NT5DC3 OSBPL10 BHLHE41 KCNK1 WDR74 EP300 KHDRBS3 BTBD3<br>PMF1 SLC43A1 INTS5 GAMT DELE1 ALDOC SH3BGRL3<br>WDR7 MPHOSPH6 PLSCR1 ACSM3 CASK MFN1 C16orf72<br>GPKOW NR1D2 ELP3HDGFL3 SSNA1 MUL1 SMURF2 BAG1<br>HOXA5 ANKFY1 SGCE ANKH SERTAD3 ID4 WDR37 RNF113A<br>RBM8A MANBA ALOX5 KIAA0100HYAL2 NKAPD1 ITPRID2 AIFM1<br>SWAP70 LBH LCN2 BRF2 ZNF33B IFT27 ZNHIT6 DNPH1<br>SAP30L WDR55 DOCK1 CALML4 VPS8 LRRC40 AKTIP ELMO2<br>MFAP3 ZBTB10 MTMR3 ASCC3 TFIP11 KATNB1 VIPAS39 HOOK2<br>RIOX1 TFF1FICD CDK5RAP1 PSMB8 CXorf40A ELP6GATAD1 CSTF2<br>NAT9 AGAP1 VEGFB GNG4 KLHL36 EMP2 ECHDC1 BCL9<br>ITPR2 C5orf30 PRKAA1 CCL2IPO8LIG3 PJA1 AVL9 SSBP2 ACADL<br>CXorf40B ERCC3 CDC73 TCEA2 MACROD1 HMOX2 COQ3<br>PDGFD ALAD CAPN5 TOX4 PFDN4 ELL3 VPS33B HLA-DQB1<br>RNF123 CDC34 CTSD ACAD8 ADM CLK3LONRF1 PER3<br>TESCTARBP1 ZNF586 NIPAL3 MYL5 PMM2 ATF2MEX3C PUDP<br>MUC1 GSTM1 RPS6KA5 PAXIP1 THTPA ISG20 MARCHF5<br>SCYL3 OVGP1 IP6K1 ANO10 TSR3TRMT13 AHCYL2 NHLRC2<br>AMFR DEAF1 MPP1 NMI ATP8B2 PCK2 RCC1L SLC7A2<br>HTRA2 EGR2 FAHD2A NPR3 PPFIBP1 CAPN7 TP53BP2 HERC5<br>RITA1 SOS1 MTOR USPL1 EPHB3 WEE1 CEP70<br>POGLUT1 ZMYM2 HCCS NAGA MAST4 KCTD5 RNF43 POR<br>ASCC1 MGAT2 LPGAT1 ING2 SNCA C1orf21 CBFA2T2 KCNN4<br>ADK HNF1B PCGF1 DNAJC16 YTHDC2 RGS17 ZNF576 SLC4A1AP

	CEBPG MCMBP ZNF239 DNAJC12 PES1ZNF281 METTL22 TOGARAM1<br>NEK3 STK25 UBIAD1 BCL10 SLC19A1 EGLN1 PPP2R1B ATXN7<br>EXOSC8 EMC8 MAP9 ARL4A RALGAPB MID1IP1 FUBP1 VARS1<br>PTPN3 SLC2A4RG N4BP2L1 ALDH4A1 LGALS8 TENT5C TMCC1<br>MAK16 EIF4ENIF1 PDCL ALAS1 PLAAT3 SP1 COQ7 RARS2<br>ASB13 HK2 TFB1M NXT2 GALNT10 TMEM62 RRP7A RFPL3<br>ATP2C2 DET1 EXOSC9 CHUK C3orf14 KLHL28 ZNF606<br>ALDH3B2 NAGLU WBP4 NSDHL CASP4 CNOT8 ACADSB FZD3<br>TMEM80 NKIRAS2 WNT5A RAB26 ICK DPM2 GREM1 ISOC2<br>NBEA BDH1 SETD6 SRRD BRMS1 PAGR1 ARMC8<br>SLC37A1 FAM174C GAS2L1 KLF11 WDR41 RFXANK PARG DALRD3<br>CCDC186 PIK3R3 PRKAG2 POLR3C ESRRA DENND2D PSMB9<br>KDM6A MT1M UBA6 ADAT1 SYT13 DDX60 MERTK COQ6<br>NOL12 ZNF140 HRAS ABHD17B ATG7 ZNF195 GNAI1 SAC3D1<br>INTS9 LSM6 DYM HOXA11 MAP3K1 ACAA2 SKP2 ZNF3<br>AKIRIN1 BYSL ALKBH1 HGSNAT C12orf4 GALNT12 CUL2 BANK1<br>VRK2 FOXN2 UVRAG CAPN1 CHN2 SPRED2 BTN3A1 SS18<br>PCOTH STN1 MGAT4B TUSC2 CSTA NAP1L2 SIPA1L3 RAB15<br>SULT2B1 CERS4 POMT1 FAH LRRC8B CXCL9 JADE3 TGDS<br>CLGN NVL RNF170 SENP2 AP5S1 HOPX BMPR1B MSH3<br>AGTPBP1 HGD TOX3 LRRC49 BRWD1 MED17 CDC14B SMARCB1<br>PATZ1 HCP5 DDA1 PHLDA2 IPO4 DOCK9 KLHL2 TBP DXO<br>SP4 TIMM22 PTK7 PSENEN STOML1 TNFAIP8 GFOD2 PLEKHB1<br>ANOS1 BID ELP5 THSD7A PKP3 ADGRA3 MAPKAP1 MAP2K5<br>GET3 NUP98 APPL1 GADD45A PANX1 THRB IFIT1<br>TMEM159 SCRG1 CDK12 IFT74 NDC1 ZNF613 TCTN2<br>LARS2 RNGTT WRN IQCK NRAS SCML1 RAP2C NR4A2<br>WDR47 RAB3IL1 NUDT15 TDRD7 SIGMAR1 RCBTB1 TNFSF13 STXBP6<br>ZNF143 CUTC MYB
red	DYNC1H1 LARS1 PNRC2 CAST SUMO1 PTOV1 SNRPD2 HDLBP<br>SF3B2 PRKACB NBR1 CYFIP1 H2AZ2 SYNGR2 NDUFA5 RANBP2<br>ERP29 SERINC3 LARP4B DLG5 ERP44 YWHAE MPDZ<br>ARMCX3 RAP1A GCSH MPHOSPH10 RSL24D1 SEC24B LRRC59<br>GLYR1 HK1 REPIN1 ABAT TACC2 NEDD8 PAIP1 ZNF292<br>ZSWIM8 MBD2 SMAD2 CUL1 PI4KA TRAPPC12 ATP6V1H<br>UBA3 SEPTIN10 ORC4 UTRN BRD7 ARFIP2 MFSD10<br>CREB3L1 WIPI2 VAPB SF1 PSMD9 FZD5 PRPF4B MAFF<br>BCAS2 POLR2J PPP1R8 RHOT2 PTPRA CLIP1 RREB1 ARFIP1<br>RIOX2 TAB2 MBD1 ZNF189 FOXN3 JOSD1 RAPGEF2 ZNF665<br>LSR XPA NEBL HEXIM1 USP24 CSNK2A1 ABI1 CEP63 KIZ<br>DNAJB12 NDUFAF3 DHX40 AKT1 PIKFYVE ELK4 RTF1 TRIM14<br>B4GALT4 NDUFS1 TMEM184C HMGXB4 RNF111 GPN3 CASP7<br>DYNC1LI1 VPS13D RNF6 HIVEP2 APOOL TMEM127 KEAP1<br>ATF1 THADA TFE3 WWOX TFDP2 DDX19A ZNF322 TIMM13 F8

	TRIP11 PDE8A MAPK14 GCDH KAT6B ELAC2 FRMD4B PPM1G<br>PPARD FEM1B WDFY3 TK2 NF2 HIPK1 DNASE1L1 SEZ6L2<br>HERC2 UTP14A PPP3R1 C11orf49 PLCB4 KDM4C NDST2 C1D<br>VPS39 TAF11 NFATC3 SEMA4D CDYL HSPA13 USP46 RNF41<br>TGIF1 MARK4 TNKS FUBP3 CDCP1

Supplementary Figure 6

5000 genes in the GSE70770，GSE32982 and GSE32269 cohort in total, and the genes were classified into eight gene modules through the WGCNA.

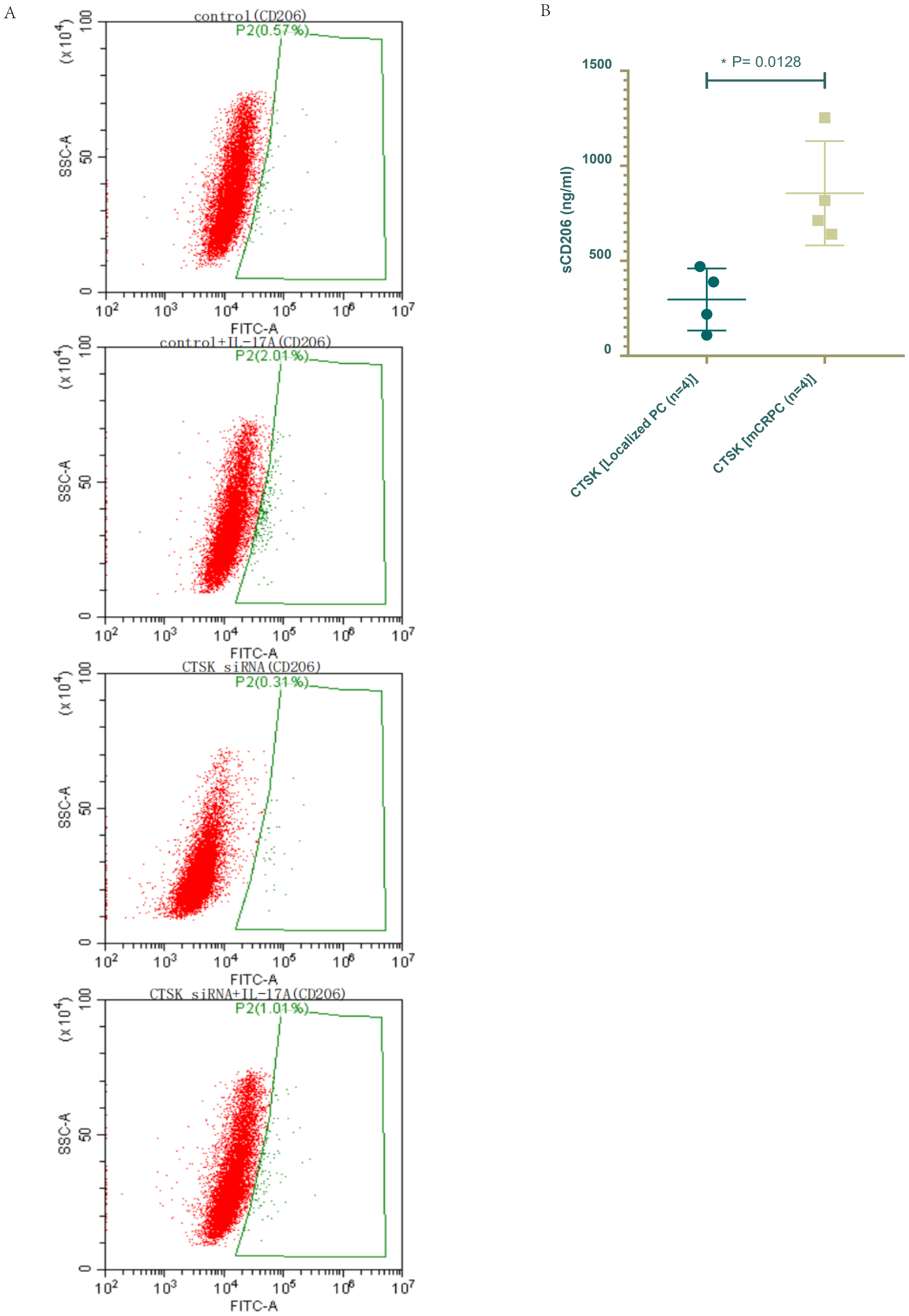

Supplementary Figure 7

(A) The proportion of immune cells infiltration in different nude mice groups was sorted by CD206. CD206 were higher in the control+IL-17A group and the CTSK siRNA+IL-17A group than the control group and the CTSK siRNA group. (B)The serum levels of sCD206 in localized PC patients and mCRPC patients. The serum levels of sCD206 in mCRPC were significantly more elevated than those in localized PC. sCD206: soluble CD206.
